# Cell-weighted polygenic risk scores are associated with β-amyloid and tau biomarkers in Alzheimer’s disease

**DOI:** 10.1093/braincomms/fcaf353

**Published:** 2025-09-12

**Authors:** Atul Kumar, Alexa Pichet Binette, Divya Bali, Shorena Janelidze, Erik Stomrud, Sebastian Palmqvist, Jacob W Vogel, Oskar Hansson, Niklas Mattsson-Carlgren

**Affiliations:** Clinical Memory Research Unit, Department of Clinical Sciences Malmö, Lund University, Malmö 211 46, Sweden; Bioinformatics Center, School of Medicine, Faculty of Health Sciences, Institute of Biomedicine, University of Eastern Finland, Kuopio 70210, Finland; Clinical Memory Research Unit, Department of Clinical Sciences Malmö, Lund University, Malmö 211 46, Sweden; Clinical Memory Research Unit, Department of Clinical Sciences Malmö, Lund University, Malmö 211 46, Sweden; Clinical Memory Research Unit, Department of Clinical Sciences Malmö, Lund University, Malmö 211 46, Sweden; Clinical Memory Research Unit, Department of Clinical Sciences Malmö, Lund University, Malmö 211 46, Sweden; Memory Clinic, Skåne University Hospital, Malmö 205 02, Sweden; Clinical Memory Research Unit, Department of Clinical Sciences Malmö, Lund University, Malmö 211 46, Sweden; Memory Clinic, Skåne University Hospital, Malmö 205 02, Sweden; Department of Clinical Sciences Malmö, SciLifLab, Lund University, Lund 22362, Sweden; Clinical Memory Research Unit, Department of Clinical Sciences Malmö, Lund University, Malmö 211 46, Sweden; Memory Clinic, Skåne University Hospital, Malmö 205 02, Sweden; Clinical Memory Research Unit, Department of Clinical Sciences Malmö, Lund University, Malmö 211 46, Sweden; Department of Neurology, Skåne University Hospital, Lund University, Lund 221 84, Sweden; Wallenberg Center for Molecular Medicine, Lund University, Lund 221 84, Sweden

**Keywords:** Alzheimer’s disease, polygenic risk score, CSF, pTau217, β-amyloid

## Abstract

The molecular pathways influencing the build-up of β-amyloid and tau pathology in Alzheimer’s disease are unclear. To investigate how the involvement of different cell types influences β-amyloid and tau, we utilized single-cell RNA-seq data to derive cell-weighted polygenic risk scores. We included participants from the BioFINDER-1 study, including cognitively unimpaired (*N* = 734) individuals and patients with mild cognitive impairment (*N* = 235), Alzheimer’s diseasedementia (*N* = 97) or non-Alzheimer’s disease neurodegenerative diseases (*N* = 227). We developed seven polygenic risk scores, including six cell-weighted (for astrocytes, excitatory neurons, inhibitory neurons, microglia, oligodendrocyte precursor cells and oligodendrocytes) and one full polygenic risk score without cell specificity. For each of the polygenic risk score models, we calculated seven scores (polygenic risk score 1–7) based on different *P*-value thresholds (ranging from *P*-value < 0.05 to *P*-value < 5e−08) of variants from an independent large Alzheimer’s disease genome-wide association study. We tested associations between the polygenic risk scores with β-amyloid [using cerebrospinal fluid (CSF) β-amyloid_1–42_/β-amyloid_1–40_], tau (using CSF pTau217) and cognitive measures (Mini-Mental State Examination score and Preclinical Alzheimer Cognitive Composite) using regression models adjusting for age, sex and the top genotype principal components. We also replicated the polygenic risk score association with β-amyloid (CSF β-amyloid_1–42_/β-amyloid_1–40_) and tau (CSF pTau217) in an independent cohort (BioFINDER-2), including cognitively unimpaired (*N* = 773) individuals and patients with mild cognitive impairment (*N* = 358), Alzheimer’s disease dementia (*N* = 286) or non-Alzheimer’s disease neurodegenerative diseases (*N* = 319). We observed differential cellular effects on β-amyloid, pTau217 and cognitive measures. There are substantial effects of neuronal-specific polygenic risk scores on β-amyloid, pTau217 and cognitive measures. The microglial-polygenic risk scores showed more significant effects on pTau217 than on β-amyloid. β-Amyloid positivity partly mediated the associations between polygenic risk scores and pTau217, with the lowest mediation effect observed for the microglial-polygenic risk scores (on average 33%). Cell-weighted gene expression has differential effects on pathological β-amyloid and tau metabolism, as well as cognitive decline. Cell-weighted gene expression related to microglia is preferentially relevant for the metabolism of soluble phosphorylated tau through partly β-amyloid-independent mechanisms. Cell-weighted gene expression related to neurons shows the strongest associations with cognition. These findings inform further studies that address specific cell types for various aspects of Alzheimer’s disease, including the development of novel treatment strategies.

## Teaser

Kumar *et al.* report distinct cellular effects on measures of β-amyloid and tau. Importantly, they also found cell-level genetic evidence for a role for microglial cells, in particular, in regulating alterations in soluble tau. This highlights the crucial role of microglia cells in dysregulated tau metabolism in Alzheimer's disease.

## Graphical Abstract

**Graphical Abstract:**
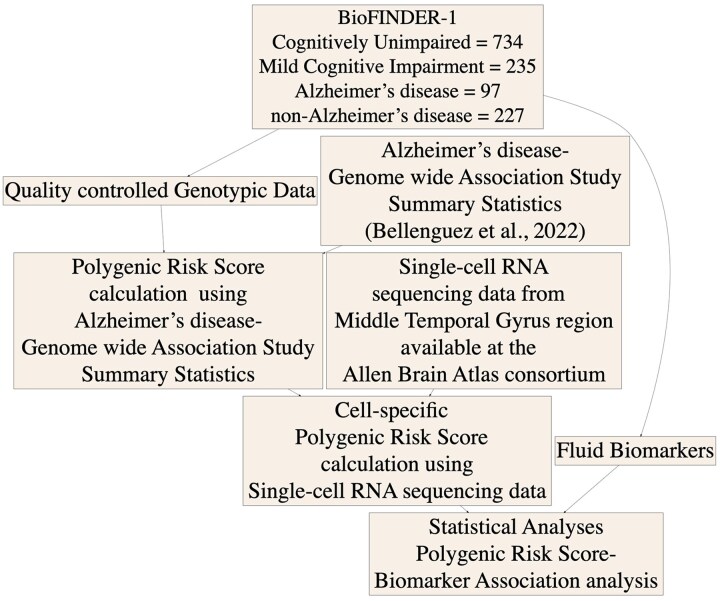

## Introduction

Alzheimer's disease is the most prevalent cause of dementia and a significant cause of death worldwide. Neuropathologic hallmarks of Alzheimer’s disease include widespread deposition of amyloid beta (Aβ) plaques and tau neurofibrillary tangles.^1,2^ Genetic studies from autosomal dominant Alzheimer’s disease and longitudinal studies in sporadic Alzheimer’s disease have established Aβ as an early event in the disease, while tangles are more intimately associated with downstream neuronal loss and clinical symptoms.^2,3^ Genome-wide association studies (GWASs) have demonstrated that sporadic, non-dominant forms of Alzheimer’s disease are also partly genetically determined,^4-6^ with hundreds of possible risk genes. Polygenic risk scores (PRSs) can enhance estimates of risk variance in a target sample and provide an individualized risk assessment. To further understand the biological pathways that affect different aspects of Alzheimer’s disease, it would be helpful if genetic risk factors could be studied in the context of different cell types. Global PRSs generated from genome-wide data do not account for the fact that different genes exhibit distinct expression patterns in various cell types. Incorporating cell-type-specific gene set PRS analysis may provide information regarding cell types or developmental phases that are biologically relevant. Gene-set-based PRS has been used to predict Alzheimer’s disease dementia in the past,^7-9^ with results generally in line with the re-analysis of Alzheimer’s disease dementia GWAS summary statistics.^4-6,10-12^ However, the association between cell-weighted genetic risk of Alzheimer’s disease and different specific Alzheimer’s disease endophenotypes has rarely been studied.^13^

Here, using single-cell RNA sequencing data from the middle temporal gyrus (MTG), available from the Allen Brain Atlas consortium (https://celltypes.brain-map.org/rnaseq), we generated cell-weighted PRS. We aimed to quantify associations of the cell-weighted Alzheimer’s disease genetic risk factors with the distinct Alzheimer’s disease endophenotype (Aβ, tau and cognitive measure) using six different cell types [astrocytes, excitatory neurons, inhibitory neurons, microglia, oligodendrocyte precursor cells (OPC) and oligodendrocytes]. Given the hypothesis that Aβ-accumulation is an initial pathological event in Alzheimer’s disease, followed by tau aggregation, we also tested to what extent the cell-weighted PRS associations with tau were mediated via Aβ.

## Materials and methods

### Standard protocol approvals, registrations and patient consent

All participants provided written informed consent before entering the study. The study procedure was approved by the local ethics committee at Lund University in Sweden [and conducted according to the Helsinki Declaration (Seventh revision (2013)].

### Study participants

The study included 734 cognitively unimpaired (CU) older adults, 235 patients with mild cognitive impairment (MCI), 97 Alzheimer’s disease dementia patients and 227 non-Alzheimer’s disease patients from the Swedish BioFINDER-1 study (clinical trial no. NCT01208675),^14^ and 773 CU, 235 MCI, 97 Alzheimer’s disease dementia patients and 227 non-Alzheimer’s disease patients from the Swedish BioFINDER-2 study (clinical trial no. NCT03174938)^15^ for whom age, gender, biomarker and cognitive measure data were available. Details on recruiting have previously been provided.^16-18^ Following research guidelines,^19^ the CU group consisted of both normal controls (*N* = 559) and patients with subjective cognitive decline (SCD) (*N* = 175) for BioFINDER-1 (Table 1) and normal controls (*N* = 554) and SCD (*N* = 219) for BioFINDER-2 (Table 2). A positive Aβ status was necessary, but not sufficient, for a clinical diagnosis of Alzheimer’s disease dementia.^20^

**Table 1:** Characteristics of the BioFINDER-1 cohort

	CU (*n* = 734)	MCI (*n* = 235)	Alzheimer’s disease dementia (*n* = 97)	Non-Alzheimer’s disease (*n* = 227)
Age	71.2 (5.8)	71.3 (5.5)	74.2 (8.2)	67.3 (8.9)
Sex (M/F)	293/441	144/91	38/59	141/86
Education	12.2 (3.5)	11.1 (3.4)	9.6 (3)	12.4 (4.1)
*APOE* ε2 (0/1/2)	628/101/5	210/25/0	90/7/0	193/34/0
*APOE* ε4 (0/1/2)	480/224/30	119/91/25	38/46/13	158/64/5
Aβ (−/+)	524/210	100/135	24/73	181/46

Age: mean (SD); *APOE* 0: allele not present, 1: heterozygous allele, 2: homozygous allele; Aβ positivity was defined as CSF Aβ_1–42_/Aβ_1–40_ < 0.091.

**Table 2:** Characteristics of the BioFINDER-2 cohort

	CU (*n* = 773)	MCI (*n* = 358)	Alzheimer’s disease dementia (*n* = 286)	Non-Alzheimer’s disease (*n* = 319)
Age	65.9 (13.6)	71.8 (8.0)	73.8 (7.4)	72.1 (8.4)
Sex (M/F)	347/426	201/157	129/157	207/112
Education	13 (3.5)	12.7 (4)	12.1 (4.1)	12.3 (3.9)
*APOE* ε2 (0/1/2)	680/91/2	317/40/1	274/12/0	276/42/1
*APOE* ε4 (0/1/2)	388/349/36	166/151/41	83/159/44	210/100/9
Aβ (−/+)	566/207	132/226	0/286	222/97

Age: mean (SD); *APOE* 0: allele not present, 1: heterozygous allele, 2: homozygous allele; Aβ positivity was defined as CSF Aβ_1–42_/Aβ_1–40_ < 0.091.

### Genotyping and preparation of genetic data

The Illumina platform GSA-MDA v2 was utilized for genotyping for BioFINDER-1 and GSA-MDA v3 for BioFINDER-2. According to established protocols, quality control (QC) was performed at the subject and single-nucleotide polymorphism (SNP) levels.^21^ Person-based quality control included consistency between chip-inferred and self-reported gender, call rates (1% cut-off) and intense heterozygosity. In addition, high-quality variants [autosomal, bi-allelic variants with Hardy–Weinberg equilibrium (HWE) *P* > 5e^−^08, minor allele frequency (MAF) ≥5% and with a call rate of > 99%] were used. More information on the imputation and quality control processes for BioFINDER has been provided previously.^22^

### Fluid biomarkers

CSF handling followed a structured pre-analytical protocol.^23,24^ CSF Aβ peptides (including Aβ1–42 and Aβ1–40) were analysed using Euroimmun immunoassays (EI) (EUROIMMUN AG, Lübeck, Germany), as previously described.^25^ A pathological Aβ status was defined as CSF Aβ1–42/Aβ1–40 < 0.091.^26^ The CSF Ptau217 assay was performed on a streptavidin small spot plate using the Meso Scale Discovery (MSD) platform (Meso Scale Discovery, Rockville, MD, USA), as previously described.^27^ For the BioFINDER-2 participants, the CSF Aβ42/40 ratio was determined through clinical measurements (Lumipulse or MSD) to assess Aβ positivity.^28^ In BioFINDER-2, analysis of CSF P-tau217 was performed at Eli Lilly and Company using the MSD platform.^27^

### Cognitive measure

The Mini-Mental State Examination (MMSE) score and modified versions of the Preclinical Alzheimer’s Cognitive Composite (mPACC)^29,30^ were used as measures for cognitive decline. MMSE is a commonly used brief cognitive test that explores five cognitive function areas: orientation, registration, attention and calculation, word recall and language, with a score ranging from 0 (worst) to 30.^31^ mPACC assesses episodic memory, timed executive function and global cognition. The total score is determined by averaging four *z*-scores, where higher scores indicate better cognitive function and lower scores indicate more cognitive impairment. mPACC has been linked to the identification of early cognitive decline in patients with Alzheimer's disease.^32^ For the longitudinal cognitive measures, individuals were followed from 2007 to 2022 (10 years on average). CUIs were followed every 2 years, and other conditions (MCI, Alzheimer’s disease and non-Alzheimer’s disease) were followed annually, resulting in an average of 5 visits per individual.

### Polygenic score calculation

To define PRSs, we utilized publicly available summary statistics from published AD GWAS studies (non-overlapping with the BioFINDER dataset).^4^ Using the weighted effect for each SNP, the PRS was determined using PLINK2.^33^ SNPs were pruned using PLINK’s clump function with an *r*^2^ < 0.1^34-36^ over 1000 kb before PRS estimation. As the *APOE* gene is the strongest genetic risk factor for sporadic Alzheimer’s disease, with high levels of linkage disequilibrium (LD) in the area surrounding the locus, we generated two PRS models: (i) including the *APOE* region and (ii) excluding the *APOE* region. In addition to observing how *APOE* status might affect the significance of the identified PRS, we also wanted to test the effect beyond *APOE*. Therefore, when generating the non-*APOE* PRS for Alzheimer’s disease, SNPs falling within the *APOE* gene region (chr19: 44905796–44909393; GRCh38 assembly) and up to 1 megabase pair^37^ upstream and downstream were omitted from the dataset. Following the standard procedure of clumping and thresholding,^38^ we iterated over a range of values (*P* < 0.05 to *P* < 5e^−08^) to maximize the predictive ability of the derived polygenic scores^39^ and generated PRS1-7 models (e.g. PRS1 includes all variants significant at *P* < 0.05; details given in Kumar *et al*.^22^). All PRS were standardized as mean = 0 and SD = 1.

### Generation of cell-weighted PRS

Using the processed single-cell RNA sequencing data from the MTG, available at the Allen Brain Atlas consortium (https://celltypes.brain-map.org/rnaseq), we generated the cell-weighted score for each gene. We used the Seurat package (version 4.3.0) and applied the function NormalizeData with the normalization.method set to *LogNormalize* and scale.factor set to 1 000 000 over the downloaded Seurat object from the Allen Brain data (raw values), Human MTG 10× SEA-AD 2022. Further, AverageExpression was applied to this log-transformed, normalized data. Using the class and subclass annotation available from the Allen Brain data, we calculated the average expression for all non-neuronal cells (microglia, astrocytes, oligodendrocytes and OPCs) and neuronal cells [GABAergic neurons (inhibitory neurons) or Glutamatergic neurons (excitatory neurons)]. The ‘None’ annotations were removed before applying AverageFunction. We then calculated a percentage expression by dividing the average expression per cell type by the sum of average expressions across all cell types.

The Human MTG 10× SEA-AD 2022 data were considered, as they include single-nucleus transcriptomes from 166 868 nuclei derived from five post-mortem human brain specimens, compared to the M1-10× genomics (2020) dataset, which includes single-nucleus transcriptomes from 76 533 nuclei derived from two post-mortem human brain specimens. Additionally, the ROSMAP data provide detailed information on gene expression changes associated with Alzheimer’s disease pathology and cognitive decline. In contrast, the Allen Brain Atlas provides a detailed view of the spatial organization of gene expression in the brain, enabling the identification of cell-type-specific gene expression patterns. Additionally, the ROSMAP data are more suitable for understanding the molecular basis of Alzheimer's disease. Conversely, the Allen Brain Atlas is more ideal for a comprehensive understanding of gene expression across various brain regions and cell types. Furthermore, Allen Brain Atlas data are from the MTG, which is one of the first brain regions to show atrophy in early Alzheimer’s disease. In contrast, the ROSMAP data from the primary motor cortex are typically affected later, with motor deficits becoming more apparent in later stages.

To generate cell-weighted PRSs, each variant was first mapped to its nearest gene using the positional mapping approach. In addition, as a sensitivity analysis, we also performed eQTL mapping by mapping SNPs to genes based on eQTL information using Functional Mapping and Annotation of Genome-Wide Association Studies (FUMA).^40,41^ eQTL mapping identified SNPs associated with genes, which likely affect their expression within a 1 Mb region (*cis*-eQTL). As eQTLs are highly tissue-specific, different brain tissues from the BRAINEAC and GTX BRAIN v6, v7 and v8 databases embedded in FUMA were selected. The default eQTL *P*-value threshold parameter was used for the mapping.

The variant weight from the summary statistics of published GWAS studies^4^ was multiplied by its corresponding mapped gene’s cell-weighted score (percentage expression across all the cell types). Thus, if a gene has a very high percentage of expression in a cell, it will be scaled to a very high value, and hence, the product of the two will be a high number. For example, variant 2:103835819 has a standardized *β* of −0.0603, and the percentage expression weight of the nearest gene, LINC01965, is 88.4% in Oligodendrocyte Precursor Cells. Then, this variant will have a scaled *β* of −5.3 in the Oligodendrocyte Precursor Cells PRS. This provided each variant with a cell-weighted weight. By implementing this methodology, an SNP that is genetically linked to a gene exhibiting a substantial effect size with Alzheimer’s disease and is highly expressed in a specific cell type will significantly influence the PRS of that cell type. Using this weighted effect for each variant, the cell-weighted PRS was determined using PLINK2,^33^ as described earlier in the method section.

An additional sensitivity analysis was performed by constructing a restricted PRS that included the genes with strict cell-specific expression. All SNPs within 1 Mb of the respective cell-specific genes were identified, and PRSs were calculated using the clumping and thresholding approach, without reweighting as described earlier.

### Statistical analyses

We used regression models to investigate the relationship between PRSs and biomarker levels. CSF ptau217 and cognitive measures were rank-based inverse normal transformed and used as dependent variables in linear regression models, adjusted for the covariates age, gender, *APOE* ε4 and ε2 counts (0, 1, 2) and the top 10 principal components (PCs) from the principal component analysis on the entire set of genotype data. Logistic regression models were used for the PRS on Aβ status (dichotomized biomarker, including the same covariates).

Mediation analysis was conducted using the R-package mediation to examine the role of Aβ in mediating the effects of cell-weighted PRS on CSF pTau217 and to assess mediation via cell-weighted average protein abundance on the associations between tau and Aβ with cell-weighted PRSs. We subtracted the coefficient for the direct effect of cell-weighted PRS (c) from the effect obtained after adjusting for Aβ, yielding the mediated effect c–c′. We used bootstrapping to estimate the 95% confidence intervals of the mediated effect^23,24^ (*n* = 1000 bootstrap samples).

The lmer function from the lme4 package was utilized to fit mixed-effect models via maximum likelihood estimation. The dependent variable for these models was the longitudinal MMSE and mPACC data. To test for the significant influence of PRS on decline, we used the following model for each cell-specific PRS


$$\begin{array}{rl} A\;<\;-\;\mathrm{lmer}(\mathrm{MMSE}/\mathrm{mPACC}\;∼ & \;\mathrm{Age}\;+\;\mathrm{Sex}\;+\;\mathrm{PC}1\;+\;\ldots \\ & +\;\mathrm{PC}10\;+\;\mathrm{PRS}\;+\;\mathrm{time}\\ & +\;\mathrm{PRS}:\mathrm{time}\\ & +\;(1\;+\;\mathrm{time}\;|\mathrm{subject}\_\mathrm{id})) \end{array}$$

$$\begin{array}{rl} B\;<\;-\;\mathrm{lmer}(\mathrm{MMSE}/\mathrm{mPACC}\;∼ & \;\mathrm{Age}\;+\;\mathrm{Sex}\;+\;\mathrm{PC}1\;+\;\ldots \\ & +\;\mathrm{PC}10\;+\;\mathrm{PRS}\;+\;\mathrm{time}\\ & +\;(1\;+\;\mathrm{time}\;|\mathrm{subject}\_\mathrm{id}))\\ & \mathrm{anova}(A,\;B) \end{array}$$

Each set of association analyses for the main analysis (98 analyses for both Aβ status and CSF pTau217 outcome) was corrected for the false discovery rate (FDR) using the Benjamini–Hochberg (BH) method. Associations below a FDR corrected *P*-value of 0.05 were considered significant.

A Welch two-sample paired *t*-test was performed to check whether the attenuation of PRSs when including/excluding *APOE* is significant. The test compares the means of two groups while considering the variability within each group. We used the formula method to obtain *t*-test results, where the *t*-values are a numerical vector and *APOE* (including/excluding) is a binary category.


t.test(t-value∼APOE(including/excluding),data)

A paired *t*-test was used as it can account for the dependency among the different PRSs. All the statistical analyses were conducted in R programming (version 4.3.0).

## Results

### Cell-weighted PRS

**Figure 1:**
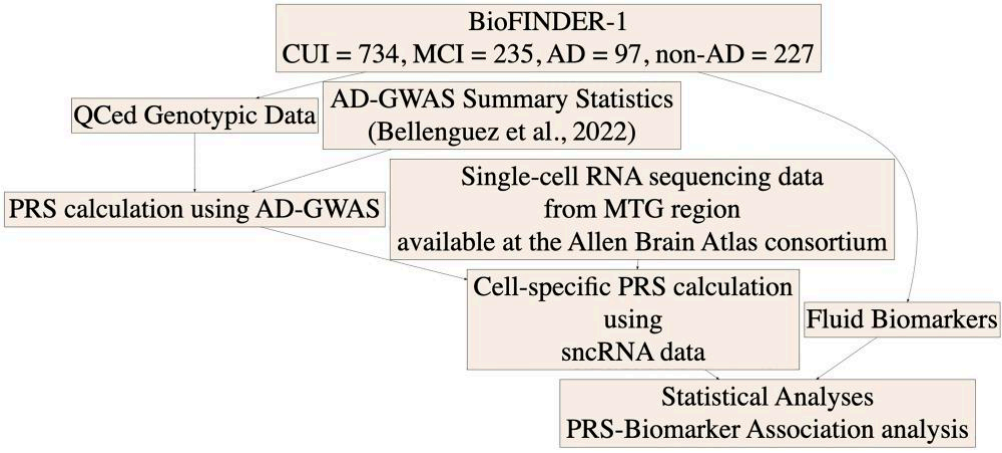
**Flow chart of the overview of the methodology.** CUI, cognitively unimpaired individual; MCI, mild cognitive impairment; AD, Alzheimer’s disease; GWAS, genome-wide association study; QCed, quality checked; PRS, polygenic risk score; MTG, middle temporal gyrus; CSF, cerebrospinal fluid; sncRNA, single-cell ribonucleic acid.

**Figure 2:**
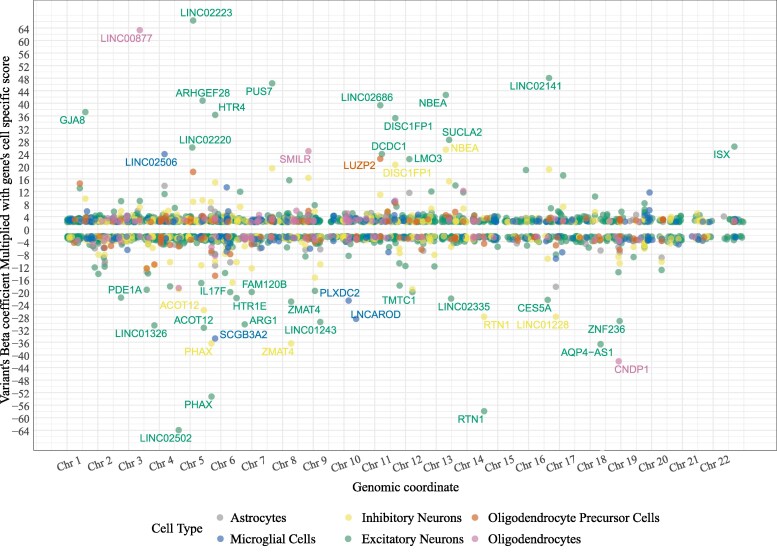
**Cell-weighted *β*-value distribution for each variant from the BioFINDER-1 genotypic data.** The *x*-axis represents the chromosomal genomic coordinates of the variants. The *y*-axis represents the variant's cell-weighted *β*-coefficient. Genes with high cell-weighted *β* (> ± 20) are named in the plot. The variants with a cell-weighted *β*-coefficient of ± 2.3 (*y*-axis 2 to −2) are not shown in the plot for the figure clarity. The variant *β*-coefficient is from the GWAS summary statistics. Each data point represents an SNP’s weighted *β* on the respective chromosome.

Using brain-derived processed single-cell RNA sequencing data, we generated six different cell-type-specific PRS. Figure 1 shows the overview of the methodology. The cell-weighted *β*-value distribution of each PRS variant is shown in Fig. 2 for different cell types. However, due to the scaling, key Alzheimer’s disease–associated genes reported in previous GWAS might not have achieved high cell-weighted *β*-values. For example, CR1 has a highest scaled weight of −4.8, CLU has a highest scaled weight of 4.2, PICALM has the highest scaled weight of 1.5, PLCG2 has the highest scaled weight of −1.9 and TREM2 has a highest scaled weight of −5.9 (Supplementary Table 1). Due to this, some cell types had larger cell-weighted SNPs (e.g. excitatory neurons), while others only had a few variants (e.g. oligodendrocytes). Supplementary Figure 1A–F shows the variants that were included in PRS7 (including the *APOE* region). In the figure, we can see that though the astrocytes are the predominant producers of *APOE* within the healthy brain, there is selective regulation of *APOE* in different cell types when considering the disease state (mainly in excitatory neurons). Supplementary Table 2 shows the descriptive statistics of the scaled *β* weights. The different cell-weighted PRSs were highly correlated for excitatory and inhibitory neurons (on average, *R* = 0.8). There was also a close relationship between neuronal PRSs and astrocytic PRSs (on average, *R* = 0.65) and between neuronal PRSs and OPC PRSs (on average, *R* = 0.6). In contrast, microglial PRSs were only weakly correlated with other cell-weighted PRSs (on average, *R* = 0.35), supporting the cell specificity of microglial PRS in particular (Supplementary Fig. 2).

### Cell-weighted PRS to predict Aβ status

**Figure 3:**
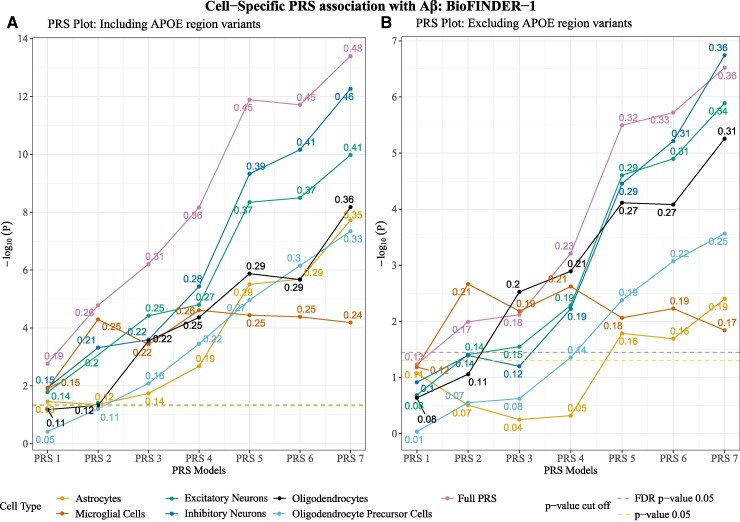
**Cell-weighted PRS association with Aβ status in BioFINDER-1.** (**A**) Cell-weighted PRS association with β-amyloid (Aβ) status for PRS models generated when including the *APOE* region variants. (**B)** Cell-weighted PRS association with Aβ status for PRS models generated when excluding the *APOE* region variants. The *x*-axis represents the respective cell-weighted PRS models, and the *y*-axis represents the negative log of the *P*-value for the association. The *β*-coefficient for the association is given at the top of each point. The models were adjusted for age, sex and the top 10 genetic principal components. PRS models excluding the *APOE* region variants were additionally adjusted for *APOE* ε4 and ε2 counts. *P*-value threshold: PRS1 => 0.05, PRS2 => 5e−03, PRS3 => 5e−04, PRS4 => 5e−05, PRS5 => 5e−06, PRS6 => 5e−07, PRS7 => 5e−08. *N* = 1293. Linear regression *β* and *P*-values are used to plot the figures. Each data point represents a −log_10_  *P*-value of the respective PRSs.

We first tested associations between cell-weighted PRS and Aβ status. The most significant association with Aβ status was found for neuronal PRSs {inhibitory neurons [*β* = 0.15–0.46 (mean: 0.3; SD: 0.12); *p*_FDR_ = 1.3e−02 to 3.8e−12] and excitatory neurons [*β* = 0.1 to 0.4 (mean: 0.3; SD: 0.1); *p*_FDR_ = 1.8e−02 to 3.6e−10]}. The microglial PRSs showed the least significant association with Aβ status [*β* = 0.15–0.24 (mean: 0.2; SD: 0.04); *p*_FDR_ = 1.4e−02 to 8.4e−05] (Fig. 3A; Supplementary Table 3A).

The associations with Aβ partly depended on the inclusion of the *APOE* region. When excluding the *APOE* region variants, an attenuation was observed for all the cell-weighted PRS models, suggesting that a part of the genetic associations with Aβ was led by genes in the *APOE* region (Fig. 3B; Supplementary Table 3B). Microglial PRSs (*t*-stat: 2.7; *P*-value: 0.02) and astrocytic PRSs (*t*-stat: 2.5; *P*-value: 0.03) had significant attenuation and became non-significant when excluding the *APOE* region, after FDR correction for multiple comparisons. Other cell-weighted PRSs showed non-significant attenuation (Supplementary Table 3C).

### Cell-weighted PRS to predict CSF pTau217

**Figure 4:**
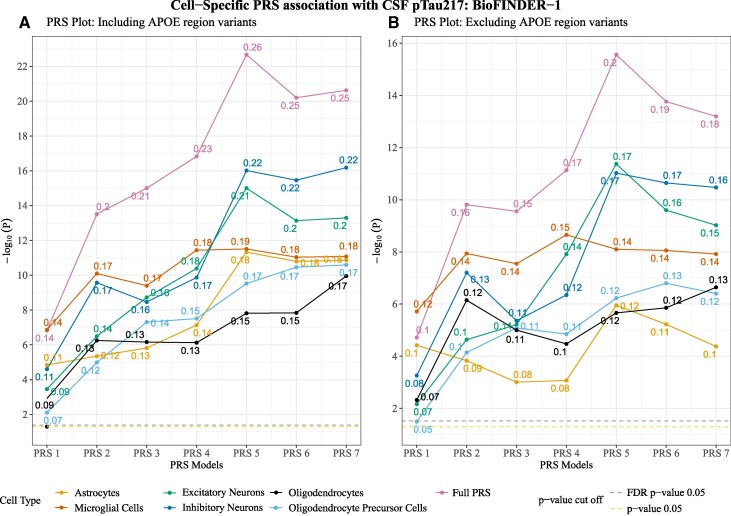
**Cell-weighted PRS association with CSF pTau217 in BioFINDER-1.** (**A**) Cell-weighted PRS association with CSF pTau217 for PRS models generated when including the *APOE* region variants. (**B**) Cell-weighted PRS association with CSF pTau217 for PRS models generated when excluding the *APOE* region variants. The *x*-axis represents the respective cell-weighted PRS models, and the *y*-axis represents the negative log of the *P*-value for the association. The *β*-coefficient for the association is given at the top of each point. The models were adjusted for age, sex and the top 10 genetic principal components. PRS models excluding the *APOE* region variants were additionally adjusted for *APOE* ε4 and ε2 counts. *P*-value threshold: PRS1 => 0.05, PRS2 => 5e−03, PRS3 => 5e−04, PRS4 => 5e−05, PRS5 => 5e−06, PRS6 => 5e−07, PRS7 => 5e−08. *N* = 1293. Linear regression *β* and *P*-values are used to plot the figures. Each data point represents a −log_10_  *P*-value of the respective PRSs.

Next, we tested the associations between cell-weighted PRS and pTau217. All cell-weighted PRSs had significant associations with pTau217. The most significant associations were found for neuronal PRSs {inhibitory neuron PRSs [*β* = 0.11–0.22 (mean: 0.2; SD: 0.04); *p*_FDR_ = 3.5e−05 to 1.3e−15] followed by excitatory neuron PRS [*β* = 0.09–0.2 (mean: 0.2; SD: 0.04); *p*_FDR_ = 4.3e−04 to 4.1e−13]} (Fig. 4A; Supplementary Table 4A). Compared with Aβ status (*t*-stat: 3.8; *P*-value: 5.5e−03), the microglial PRSs were more significantly associated with pTau217 [*β* = 0.14–0.18 (mean: 0.2; SD: 0.02); *p*_FDR_ = 2.8e−07 to 4.1e−11] and sometimes outperformed the neuronal PRSs in terms of significant association with pTau217 (PRS-2 to PRS-4) (Supplementary Table 4B).

After excluding the *APOE* region variants, the association with pTau217 for all the cell-weighted PRSs was attenuated, suggesting that *APOE* region genes led a part of the association with pTau217 (Fig. 4B; Supplementary Table 4C). However, the attenuation was relatively low compared with Aβ associations (Supplementary Table 4D), and all the PRS models remained significant after FDR correction for multiple comparisons. Among all the cell-weighted PRSs, the most considerable attenuation was for microglial PRS (*t*-stat: 4.9; *P*-value: 7e−04), astrocyte PRSs (*t*-stat: 4; *P*-value: 3.5e−03) and inhibitory neurons PRSs (*t*-stat: 2.3; *P*-value: 3.9e−02) and was non-significant for excitatory neurons PRSs (*t*-stat: 1.9; *P*-value: 8.4e−02), OPC PRSs (*t*-stat: 2.1; *P*-value: 6.1e−02) and oligodendrocytes PRSs (*t*-stat: 2.1; *P*-value: 5.7e−02) (Supplementary Table 4E).

### Cell-weighted PRS to predict CSF pTau217 when adjusting for Aβ status

**Figure 5:**
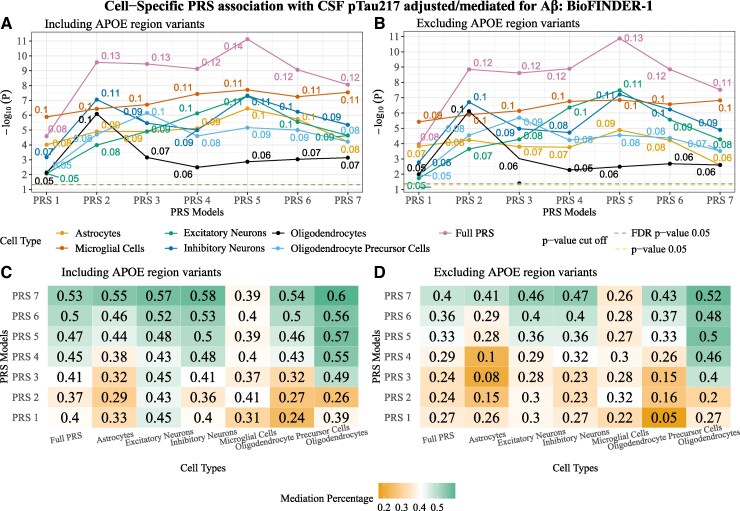
**Cell-weighted PRS association with CSF pTau217 adjusted/mediated for Aβ status in BioFINDER-1.** (**A**) Cell-weighted PRS association with CSF pTau217 adjusted for Aβ status for PRS models generated when including the *APOE* region variants. (**B**) Cell-weighted PRS association with CSF pTau217 adjusted for Aβ status for PRS models generated when excluding the *APOE* region variants. The *x*-axis represents the respective cell-weighted PRS models, and the *y*-axis represents the negative log of the *P*-value for the association. The *β*-coefficient for the association is given at the top of each point. The models were adjusted for age, sex, Aβ status and the top 10 genetic principal components. PRS models excluding the *APOE* region variants were additionally adjusted for *APOE* ε4 and ε2 counts. *P*-value threshold: PRS1 => 0.05, PRS2 => 5e−03, PRS3 => 5e−04, PRS4 => 5e−05, PRS5 => 5e−06, PRS6 => 5e−07, PRS7 => 5e−08. Linear regression *β* and *P*-values are used to plot the figures. Each data point represents a −log_10_  *P*-value of the respective PRSs. (**C**) Heat map of Aβ mediation for CSF pTau217 association with PRS models generated when including the *APOE* region variants. (**D**) Heat map of Aβ mediation for CSF pTau217 association with PRS models generated when excluding the *APOE* region variants. The *x*-axis represents the different cell types, and the *y*-axis represents the PRS models for respective cell types. *N* = 1293.

Under the hypothesis that pTau217 increases in response to Aβ pathology, we considered the possibility that Aβ status confounded the associations between PRS and pTau217. We tested if cell-weighted PRSs were associated with pTau217 when adjusting for Aβ status (Fig. 5A; Supplementary Table 5A). We found significant attenuation for all cell-weighted PRSs compared to models that did not adjust for Aβ status; however, the associations between cell-weighted PRSs and CSF pTau217 remained significant. Among all the cell-weighted PRSs, the most considerable attenuation was observed for microglial PRSs (*t*-stat: 10.5; *P*-value: 1.1e−05), followed by oligodendrocyte PRS (*t*-stat: 6; *P*-value: 1.3e−04), and neuronal PRSs [inhibitory neurons PRSs (*t*-stat: 5.3; *P*-value: 9.9e−04) and excitatory neurons PRS (*t*-stat: 4.5; *P*-value: 1.7e−03)] (Supplementary Table 5B).

After excluding the *APOE* region variants and adjusting for Aβ (Fig. 5B; Supplementary Table 5C), all the cell-weighted PRSs showed significant attenuation when compared with when not adjusting for Aβ. The most considerable attenuation was observed for microglial PRSs (*t*-stat: 10.8; *P*-value: 4.4e−06), followed by oligodendrocyte PRS (*t*-stat: 4.8; *P*-value: 5.1e−04) (Supplementary Table 5D).

### Aβ status mediates the effects of cell-weighted PRS on CSF pTau217

Again, under the hypothesis that pTau217 increases in response to Aβ, we tested whether Aβ status mediated the associations between cell-weighted PRSs and pTau217. A significant mediation effect of Aβ (24–60%) was observed on all the cell-weighted PRSs associated with CSF pTau217 (Fig. 5C; Supplementary Tables 6–12).

Results were similar when excluding the *APOE* region variants. However, the mediation effect of Aβ on PRSs with a large number of variants (PRS1-2) became non-significant for most of the cell-weighted PRSs (Fig. 5D; Supplementary Tables 13–19).

### Interactions between Aβ status and cell-weighted PRS to predict CSF pTau217

To formally test if the effect of cell-weighted PRS association with pTau217 differed by Aβ status, the analyses were further adjusted for Aβ–PRS interaction. This interaction was significant for inhibitory and excitatory neurons (PRS7), supporting that the genetic variants related to these cell types have differential effects on pTau217 depending on the presence or absence of Aβ (Supplementary Fig. 3A and Table 20A).

When the *APOE* region variants were excluded, Aβ–PRS interaction for inhibitory and excitatory neurons (PRS7) remained significant (Supplementary Fig. 3B and Table 20B).

### Cell-weighted PRS to predict cognitive decline

**Figure 6:**
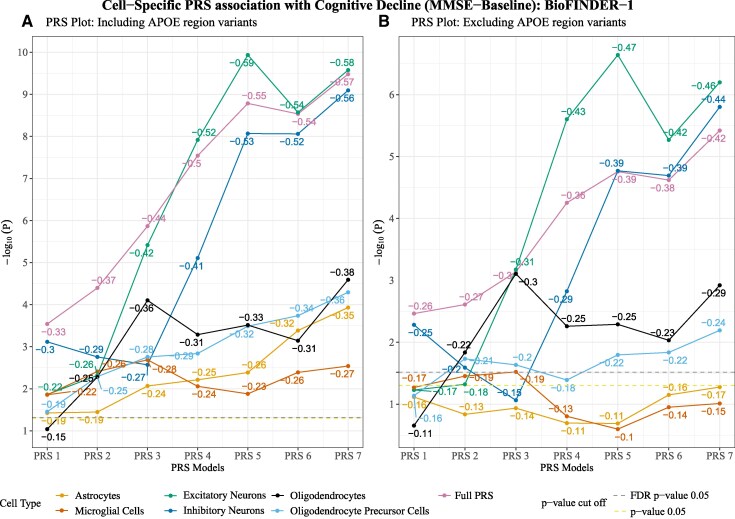
**Cell-weighted PRS association with baseline cognitive measure (MMSE) in BioFINDER-1.** (**A**) Cell-weighted PRS association with Mini-Mental State Examination (MMSE) for polygenic risk score (PRS) models is generated when the *APOE* region variants are included. (**B**) Cell-weighted PRS association with MMSE for PRS models generated when excluding the *APOE* region variants. The *x*-axis represents the respective cell-weighted PRS models, and the *y*-axis represents the negative log of the *P*-value for the association. The *β*-coefficient for the association is given on the top of each bar. The models were adjusted for age, sex and the top 10 genetic principal components. PRS models excluding the *APOE* region variants were additionally adjusted for *APOE* ε4 and ε2 counts. *P*-value threshold: PRS1 => 0.05, PRS2 => 5e−03, PRS3 => 5e−04, PRS4 => 5e−05, PRS5 => 5e−06, PRS6 => 5e−07, PRS7 => 5e−08. *N* = 1293. Linear regression *β* and *P*-values are used to plot the figures. Each data point represents a −log_10_  *P*-value of the respective PRSs.

Using baseline and longitudinal data, we also tested the cell-weighted PRS association with cognitive measures (MMSE and mPACC). For the baseline cognitive measure (MMSE), neuronal PRSs {excitatory neuron PRSs [*β* = −0.2 to −0.6 (mean: −0.45; SD: 0.15); *p*_FDR_ = 1.5e−02 to 5.4e−09] followed by inhibitory neuron PRS [*β* = −0.3 to −0.6 (mean: −0.4; SD: 0.12); *p*_FDR_ = 1.4e−03 to 9.8e−09]} showed the strongest association. The microglial PRSs showed the weakest association with baseline MMSE [*β* = −0.2 to −0.3 (mean: −0.25; SD: 0.02); *p*_FDR_ = 1.5e−02 to 4.3e−03] (Fig. 6A; Supplementary Table 21A).

Similar to Aβ and pTau217, after excluding the *APOE* region variants, the association with baseline MMSE for microglial PRSs (*t*-stat: −6.6; *P*-value: 5e−05), astrocytes PRSs (*t*-stat: −4.8; *P*-value: 1.5e−03) and OPC PRSs (*t*-stat: −3.6; *P*-value: 6.7e−03) had significant attenuation and became non-significant when excluding the *APOE* region, after FDR correction for multiple comparisons. Other cell-weighted PRSs showed non-significant attenuation (Fig. 6B; Supplementary Table 21B and C).

With baseline mPACC, neuronal PRSs {inhibitory neuron PRSs [*β* = −0.09 to −0.18 (mean: −0.15; SD: 0.03); *p*_FDR_ = 2.5e−02 to 1.7e−05] and excitatory neuron PRS [*β* = −0.04 to −0.16 (mean: −0.12; SD: 0.04); *p*_FDR_ = 3.2e−01 to 7.0e−05]} followed by the microglial PRSs [*β* = −0.1 to −0.14 (mean: −0.12; SD: 0.02); *p*_FDR_ = 7.1e−03 to 4.1e−04] showed substantial associations. After correcting for multiple comparisons, remaining cell-weighted PRSs showed non-significant associations with baseline mPACC (Supplementary Fig. 4A and Table 22A).

When excluding the *APOE* region variants, a significant attenuation in the association with baseline mPACC was observed for astrocytes PRSs (*t*-stat: −3.2; *P*-value: 7.8e−03), microglial PRSs (*t*-stat: −2.5; *P*-value: 0.03) and inhibitory neuron PRSs (*t*-stat: −2.3; *P*-value: 0.04). Other cell-weighted PRSs showed non-significant attenuation (Supplementary Fig. 4B and Table 22B and C).

However, none of the cell-weighted PRSs showed a significant association with longitudinal cognitive measures (MMSE and mPACC) (Supplementary Tables 23A and B and 24A and B).

Compared with the cell-weighted PRSs, full PRSs (PRSs without any correction for cell-weighted score) showed the strongest association with cognitive measures as well as Aβ status (Fig. 3A and B) and CSF pTau217 (Fig. 4A and B).

### Comparing the result of positional versus eQTL mapping

We performed eQTL mapping by mapping SNPs to genes based on the eQTL information using FUMA to compare how an alternative gene mapping method might affect the results. Cell-specific PRSs (including the *APOE* region) were generated using eQTL-mapped genes and tested for their association with Aβ and CSF ptau217. Comparing the results of eQTL mapping and positional mapping, we found no significant difference in results, and the positional mapping results still hold (Supplementary Fig. 5A and B).

### Restricted PRS analysis

We also performed a restricted PRS analysis to test if the results vary based on the methodological changes. Testing the association of CSF pTau217 with restricted PRS resulted in an attenuated PRS effect for all the cell-specific PRSs, but still showed a significant effect for microglial, excitatory and inhibitory neuron PRSs both while including and excluding *APOE* (Supplementary Fig. 6A and B).

### Replication of cell-weighted PRS association with Aβ status and CSF Ptau217 in an independent cohort

To determine whether our findings were not due to the characteristics of a particular cohort, we tested the cell-weighted association with Aβ status and CSF Ptau217 from an independent sample: the BioFINDER-2 cohort, which does not overlap with the BioFINDER-1 cohort. Cell-weighted PRS association with Aβ status, cell-weighted PRS association with CSF pTau217 and cell-weighted PRS to predict CSF pTau217 when adjusting for Aβ status were successfully replicated in BioFINDER-2 showing similar results as in BioFinder-1 (Supplementary Figs 7A, B, 8A, B and 9A, B and Tables 25–28). The mediation effect of Aβ on the association of cell-weighted PRS with CSF pTau217 was also replicated in BioFinder-2. It showed a higher percentage of mediation effect of Aβ (60–90%) compared with BioFinder-1 (Supplementary Fig. 9C and D). Replicating if the effect of cell-weighted PRS association with pTau217 differed by Aβ status, we observed that interaction was only significant for microglial PRSs and excitatory neurons (PRS6). Unlike the original finding, we could not identify a significant interaction term for inhibitory neurons (Supplementary Fig. 10A and B). The detailed result is provided in the Supplementary material.

## Discussion

We established links between cell-type-specific AD-PRSs and two prominent pathological Alzheimer’s disease features, Aβ and phosphorylated tau biomarkers, measured in living humans. We found that neuronal PRSs are more strongly associated with Aβ status than microglial and other neuroglial cell PRSs. On the other hand, microglial PRSs and astrocytic PRSs were strongly associated with CSF pTau217. In addition, the cell-weighted association with pTau217 was partly mediated by Aβ, especially for neuronal PRS, and less so for microglial PRS. We also successfully replicated these findings in an independent cohort.

Using the *P*-value thresholding approach, we have found that PRS generated using SNPs with a more stringent *P*-value (PRS 5, 6 or 7) have shown a significant association with endophenotypes compared with PRS generated using a relaxed *P*-value (e.g. PRS1). Previous studies^9,42^ have also demonstrated a similar pattern where a PRS with a stringent *P*-value is strongly associated with the endophenotype. In contrast, other studies^43,44^ have shown a strong association between the PRS generated with a relaxed *P*-value and the endophenotypes. A possible explanation for such contradictory findings might be the choice of endophenotype and the different confounding factors used in a particular study.^45^

The association between Aβ and cell type-specific PRS, particularly its substantial link with neuronal PRSs and relatively less association with other PRSs, is intriguing and in agreement with a large body of literature that links Aβ-production and potential aggregation to the activity of neurons and synapses.^46-48^ According to one of these studies, synaptic activity directly and dynamically influences the levels of Aβ in the brain's interstitial fluid over minutes to hours.^46^ A separate study suggests that neuronal impairment affecting memory function in specific brain areas is linked to amyloid pathology. This indicates that the early stages of Alzheimer’s disease may be present in cognitively intact older adults exhibiting signs of amyloid pathology.^47^ An additional study revealed that the precuneus, medial orbitofrontal and posterior cingulate cortices are where Aβ build-up predominantly begins.^48^

One important finding in our study was the preferential association between pTau217 and microglial PRSs. This association was only a minor part affected by adjusting for Aβ. The observation that genetic activity relevant to microglial activity can lead to differential levels of pTau217 is a novel and important finding. Our statistical mediation analyses further support the notion that the relationship between cell-weighted PRSs and pTau217 levels may exhibit varying degrees of independence from Aβ, depending on the cell type. The low degree of mediation observed for microglial PRSs suggests that the link between gene expression in microglia and increased pTau217 levels does not involve an Aβ intermediate step (although there may, of course, be other mechanisms not examined here, beyond Aβ, that may mediate the effect of microglial PRS on pTau217). We found an interaction between Aβ and neuronal PRSs on pTau217, suggesting that neuronal molecular activity has a differential impact on pTau217 depending on whether Aβ is present.

In contrast, there were no such interaction effects for microglial PRSs, supporting that microglia's molecular activity is closely linked to pTau217 rather than Aβ. Microgliosis is an important aspect of Alzheimer’s disease, with activated microglia associated with Aβ-plaque deposition.^49,50^ Taken together, our findings on both Aβ and pTau217 are consistent with a scenario where Aβ-deposition is initiated due to molecular processes that largely involve neuronal activity, but the (downstream) development of tau pathology depends more on molecular processes that involve microglial activity.^51,52^ Therefore, this study's findings contribute to the understanding of the relationship between Aβ pathology, microglial regulation and altered tau metabolism. The significant association between CSF pTau217 levels and both neuronal and glial cell PRSs is in agreement with earlier literature, which shows roles for both neuronal and glial cells in tauopathies.^53,54^ The literature review emphasized the functional implications of tau overexpression in glial cells and investigated the potential role of glial tau pathology in developing neurodegenerative tauopathies.^53^ One recent study that used the mouse model discovered that suppression of microglia or T cells prevented tau-mediated neurodegeneration and that mice with tauopathy, but not those with amyloid accumulation, acquired a distinct innate and adaptive immune response.^54^ To our knowledge, only one previous study has tested the effects of cell-type PRSs on Alzheimer’s disease endophenotypes (using ROSMAP and A4 study data, with neuropathology and PET data).^13^ That study suggested that microglia are involved in the late stages of plaque maturation and in the formation of neurofibrillary tangles and the aberrant accumulation of tau protein. Our study agrees with microglia’s role in influencing abnormal tau metabolism in the form of altered levels of soluble CSF p-tau127.^55^ According to the study,^55^ microglial activation slowed down the neocortical Braak III–VI phases of the disease when tau accumulation, which is closely linked to the onset of dementia, depends on cortical amyloid-β aggregates. A critical difference between our studies is that the previous study^13^ did not examine CSF biomarkers or any measure of ptau217, which is a sensitive marker for altered tau metabolism in Alzheimer’s disease.^56^ Another difference is that all the PRSs in Yang *et al*.^13^ were constructed by excluding the *APOE* region SNPs. In contrast, this study presents associations when including and excluding *APOE* region SNPs, illustrating the relative effects of genetic variants beyond those related to *APOE*.

After removing all SNPs in the *APOE* region and within 1 Mp upstream and downstream of the *APOE* region, the impact of the PRSs was diminished, and (accounting for multiple testing) none of the astrocytic-weighted PRS associations remained statistically significant for Aβ, but all were significant for CSF pTau217 (though considerably attenuated) (Supplementary Tables 3 and 6). This supports an astrocyte-specificness of *APOE*.^57,58^ The significance of the *APOE* region in contributing a substantial portion of the association signal for the cell-weighted PRS models was anticipated, given the well-established strong relationship between the *APOE* locus and the risk of Alzheimer’s disease,^59,60^ especially for Aβ pathology.^61^ The association is particularly pronounced in populations mostly of European descent,^62^ which is consistent with the population under study in our research. Nevertheless, the cell-weighted PRSs exhibited associations with CSF pTau217 levels that extended beyond the influence of the *APOE* locus. This aligns with the hypotheses that genetic factors affecting tau metabolism are largely not *APOE*-related.

In this study, we used the nearest gene approach to map the variants to the causal gene. Although various approaches exist for variant mapping, few previous studies^63,64^ have shown the nearest gene approach to be among the top methods for gene prioritization. One of these studies^63^ demonstrated that the nearest gene approach, along with Data-driven Expression-Prioritised Integration for Complex Traits (DEPICT),^65^ achieves comparable precision and recall to other methods. Other study^64^ using the polygenic priority score (PoPS) for gene prioritization showed that the PoPS and the nearest gene approach individually outperform other gene prioritization methods. They also observed the best overall performance by combining PoPS with orthogonal methods. Another study,^66^ combining data from GWAS and whole-exome sequencing (WES), showed an enrichment of association of GWAS loci when focusing only on genes nearest to GWAS sentinel variants, including those for non-coding risk loci. Despite being a common choice for gene mapping, the nearest gene approach has the drawback of being more suitable for GWAS-significant loci but possibly not the best for SNPs with less significant *P*-values. Additionally, the positional mapping approach employed in the study does not account for situations where a single genetic signal involves a locus containing multiple genes, each of which could be a potential candidate, nor does it consider potential trans-effects, where a genetic variant influences genes located far from the identified locus. To determine if these factors affect our findings, we conducted a sensitivity analysis by mapping SNPs to genes based on the eQTL information, which confirms that there is no significant difference in our findings.

The additional sensitivity analysis, which restricts the PRS to include only genes with strict cell-specific expression, attenuated the effects of the PRS. Due to the non-weighing of SNP effects by cell-specific gene, as some genes exhibit extremely high expression in one cell type compared to other cell types, this might have markedly diluted the effects of the PRSs and rendered the study much less informative.

Our methodology substantially expands upon and enhances previous techniques for assessing the cell type specificity in the heredity of dementia.^4,67^ In a broader sense, our work supports the benefits of using cell-weighted data for improved genomic prediction. However, further research is required to determine the extent to which this improvement can be replicated and whether it has sufficient therapeutic significance.

Several limitations should be taken into consideration. Due to inherent biological complexities, incorrect SNP assignment to genes can significantly impact the advantage of cell-specific PRS. As a result, the outcome of such studies should be interpreted with care in the light of such potential sources of error. The scope of the study was limited to persons of European ancestry due to the availability of well-powered AD GWAS summary statistics exclusively from this population. The generalizability of our findings to populations of different ancestries remains uncertain. Furthermore, experimental studies focusing on specific genes (e.g. BIN1)^68^ have shown that genetic variants may differ in their effects on gene expression, despite similar overall levels of gene expression across different cell types.

Notwithstanding the aforementioned limitations, our research utilized extensively described datasets to uncover strong and consistent causal relationships between genetic risk factors for dementia, which are localized to various cell types, and diverse pathological mechanisms underlying the development of dementia. Furthermore, as demonstrated by our and other studies,^69-71^ applying cell-type-specific PRS approaches may facilitate the development of a mechanistic understanding of the structural alterations associated with polygenic risk in Alzheimer’s disease. Additionally, it can be expanded to encompass phenotypes beyond various forms of dementia, provided that accessible GWAS summary statistics and well-defined target datasets are available. Applying our approach to analyse individual tissue types could also be advantageous. In the future, there is potential for studies that integrate cell-type-specific polygenic methodologies with extensive multimodal data obtained from thoroughly phenotyped cohorts and model systems. Additional studies involving several cell states, sometimes referred to as subtypes, encompassing disease-associated states,^72,73^ might also provide insight into disease mechanisms at the cellular level. Such studies have the potential to provide a more comprehensive understanding of the causal factors underlying the cellular mechanisms involved in dementia development.

## Supplementary Material

fcaf353_Supplementary_Data

## Data Availability

Anonymized data will be shared upon request from a qualified academic investigator for the sole purpose of replicating procedures and results presented in the article and providing that the data transfer is in agreement with European Union legislation on the general data protection regulation and decisions by the Ethical Review Board of Sweden and Region Skåne, which should be regulated in a material transfer agreement. The code used in this study is available at https://github.com/atulkumar1301/Cell_Specific_PRS.
