# Supplementary material for: Cell-weighted polygenic risk scores are associated with β-amyloid and tau biomarkers in Alzheimer’s disease
Source: Brain Commun. 2025 Sep 12;7(5):fcaf353. doi: 10.1093/braincomms/fcaf353 (PMC12484444; doi:10.1093/braincomms/fcaf353)
Supplement: fcaf353_Supplementary_Data [file fcaf353_supplementary_data.zip › Supplementary_Material.pdf]

Cell-specific polygenic risk scores are associated with Aβ and tau biomarkers in Alzheimer's disease

Atul Kumar, Alexa Pichet Binette, Divya Bali, Shorena Janelidize, Erik Stomrud, Sebastian Palmqvist, Jacob W. Vogel, Oskar Hansson, Niklas Mattsson-Carlgren

Supplementary Result

Replication of cell-specific PRS association with Aβ status and CSF P-tau217 in an independent cohort

To determine whether our findings were not due to the characteristics of a particular cohort, we tested the cell-specific association with Aβ status and CSF P-tau217 from an independent sample: the BioFINDER-2 cohort, which does not overlap with the BioFINDER-1 cohort.

Cell-specific PRS association with Aβ status in BioFINDER-2

We replicated the associations between cell-specific polygenic risk scores (PRS) and Aβ status. Like BioFINDER-1 the most significant association with Aβ status was found for neuronal PRSs (Inhibitory Neurons [β=0.2-0.5 {mean:0.4; SD: 0.12}; $p_{Bonferoni}$=9.6e-05-3.4e-20 {effective p-value=3.5e-82}] and Excitatory Neurons [β=0.2-0.4 {mean:0.3; SD: 0.1}; $p_{Bonferoni}$=1.1e-04-7.6e-16 {effective p-value=1.6e-61}]) and OPC PRSs [β=0.18-0.45 {mean:0.4; SD: 0.1}; $p_{Bonferoni}$=5.9e-04-2.3e-16 {effective p-value=2.5e-63}]. The microglial PRSs showed the least significant association with Aβ status [β=0.1-0.18 {mean:0.16; SD: 0.03}; $p_{Bonferoni}$=6.5e-02-2.5e-04 {effective p-value=6.2e-14}] (Supplementary Figure 7A).

Similar to BioFINDER-1, the associations with Aβ partly depended on including the *APOE* region. Microglial PRSs (t-stat: 2.7; p-value: 0.02), Astrocytes PRSs (t-stat: 2.5; p-value: 0.03) and Oligodendrocyte PRSs (t-stat: 2.4; p-value: 0.03) had the significant attenuation and became non-significant when excluding the *APOE* region, after Bonferroni correction for multiple comparisons. Other cell-specific PRSs showed non-significant attenuation (Supplementary Figure 7B; Supplementary Table 9).

Cell-specific PRS association with CSF pTau217 in BioFINDER-2

The cell-specific PRS association with pTau217 was also very well replicated in the BioFINDER-2 cohort. Similar to BioFINDER-1, the most significant associations were shown by neuronal PRSs (Inhibitory Neuron PRSs [β=1.7-4.6 {mean:3.5; SD: 1.1}; $p_{Bonferoni}$=2.1e-03-4.2e-16 {effective p-value=9.3e-61}] followed by Excitatory Neuron PRS [β=1.6-4.1 {mean:3.1; SD: 1.1}; $p_{Bonferoni}$=6.1e-03-3.3e-13 {effective p-value=8e-48}] (Supplementary Figure 8A).

After excluding the *APOE* region variants, the most considerable attenuation was for astrocyte PRSs (t-stat: 3.3; p-value: 7.6e-03), OPC PRSs (t-stat: 2.2; p-value: 0.05) and Oligodendrocytes PRSs (t-stat: 2.2; p-value: 0.05) (Supplementary Figure 8B; Supplementary Table 10).

Cell-specific PRS to predict CSF pTau217 when adjusting for Aβ status in BioFINDER-2

We also tested if cell-specific PRSs association with pTau217 when adjusting for Aβ-status still hold in the independent cohort (Supplementary Figure 9A). The results matched the original findings and showed a significant attenuation for all the cell-specific PRSs compared to models without adjusting for Aβ-status. (Supplementary Table 11).

The results were the same when excluding the *APOE* region variants. All the cell-specific PRSs showed significant attenuation, making the cell-specific PRS association with CSF pTau217 non-significant (Supplementary Figure 9B) (Supplementary Table 12).

Aβ status mediates the effects of cell-specific PRS on CSF pTau217

We also tested to confirm whether Aβ mediates cell-specific PRSs' associations with pTau217. Similar to the original findings, a significant and considerable mediation effect of A*β* (60-90%) was observed on all the cell-specific PRSs associated with CSF pTau217 (Supplementary Figure 9C).

Similar results were observed when excluding the *APOE* region variants (Supplementary Figure 9D).

Interactions between Aβ status and cell-specific PRS to predict CSF pTau217

Finally, to test and replicate if the effect of cell-specific PRS association with pTau217 differed by Aβ-status, we adjusted the model for Aβ-PRS interaction. We observed that interaction was

only significant for microglial PRSs and excitatory neurons (PRS-6). Unlike the original finding, we could not find a significant interaction term for inhibitory neurons.

We also observed a similar result when excluding the *APOE* region variants where Aβ-PRS interaction for microglial and excitatory neurons PRSs remained significant (Supplementary Figure 10A-B).

Supplementary Figure

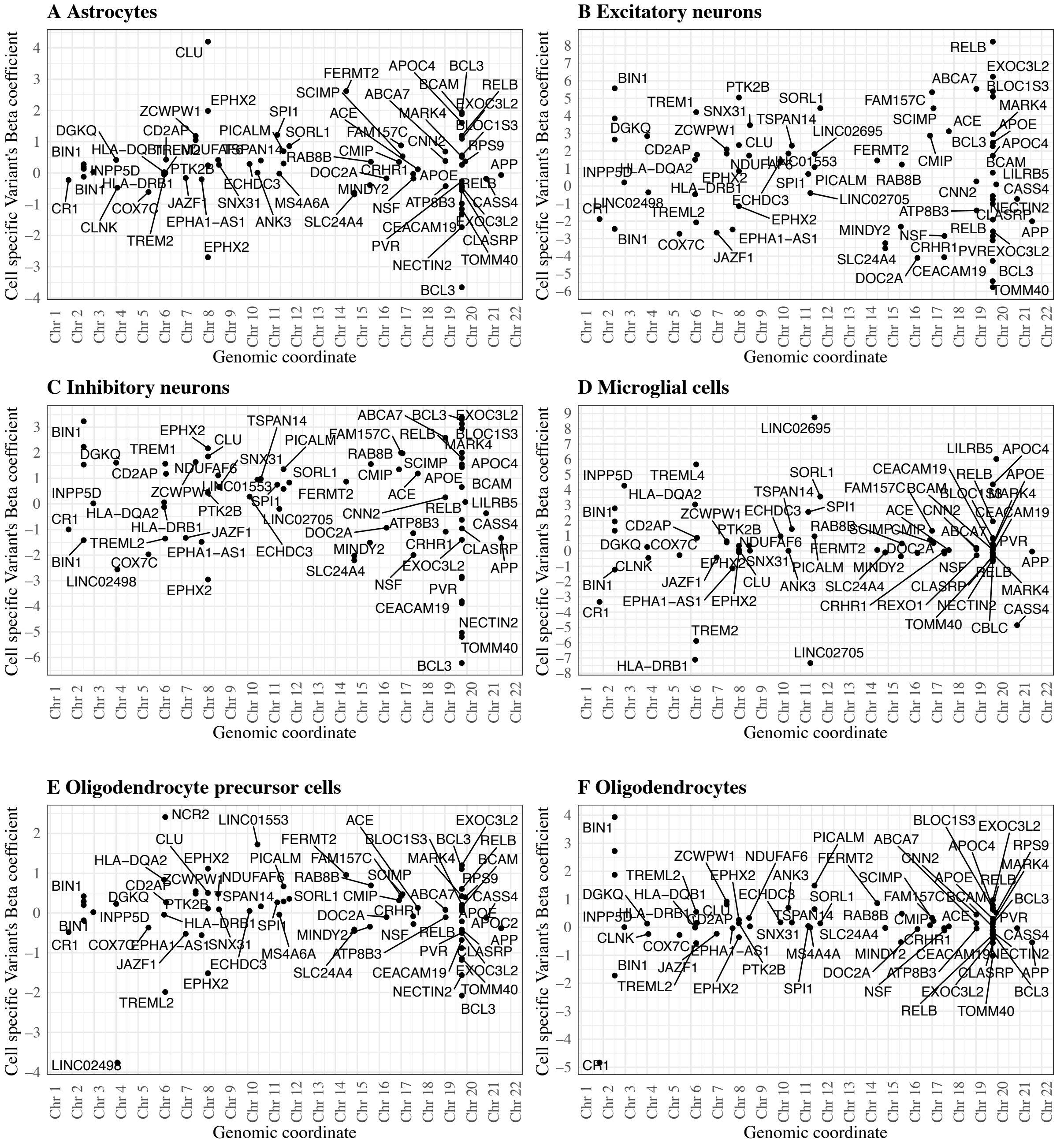

Supplementary Figure 1: Cell-weighted beta value distribution for each variant in PRS7 from the BioFINDER-1 genotypic data. The X-axis represents the chromosomal genomic coordinates of the variants. The Y-axis represents the Variant's cell-weighted β-coefficient. The variants β-coefficient is from the GWAS summary statistics. Each data point represents a SNP's weighted beta on the respective chromosome.

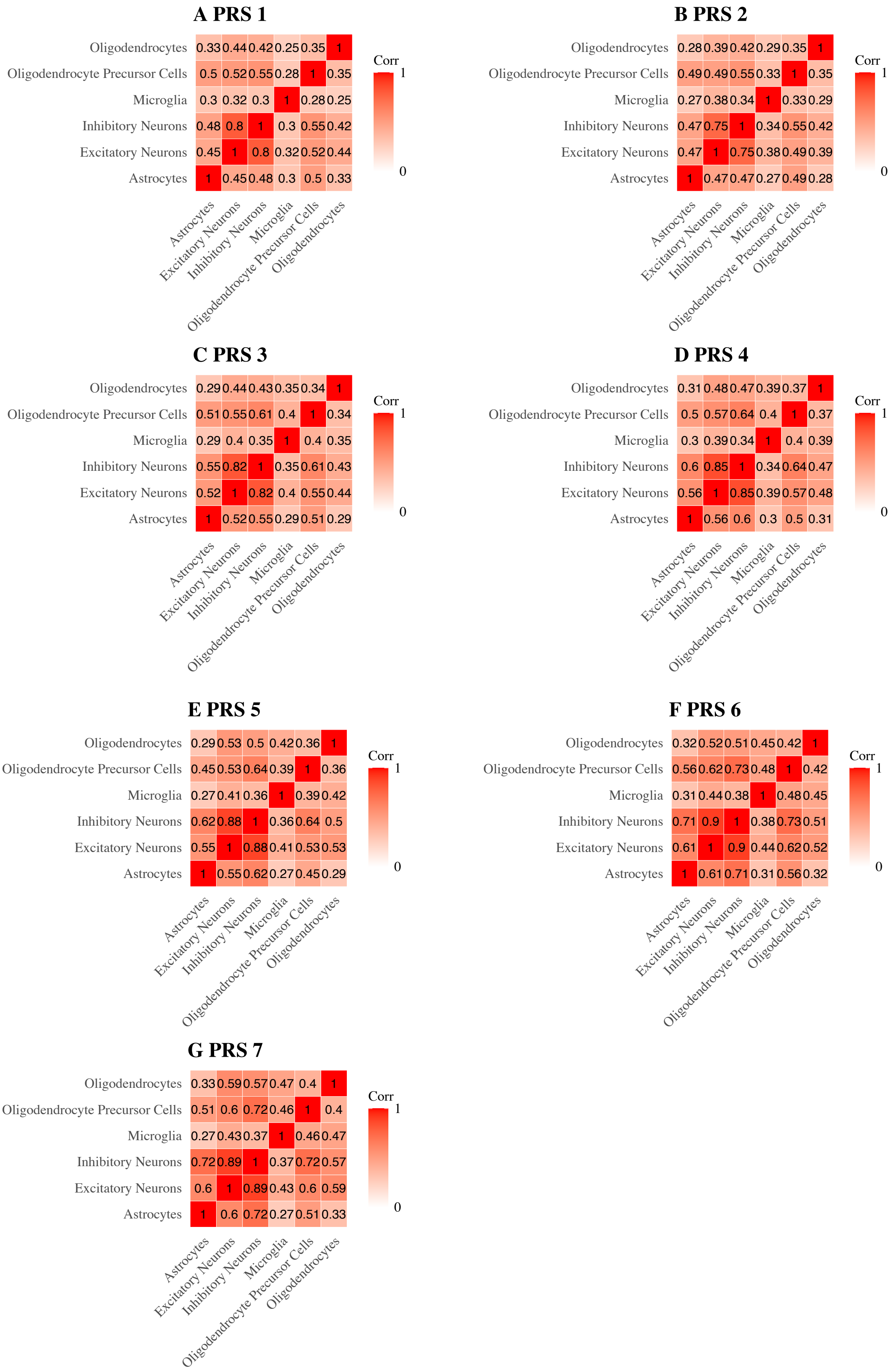

Supplementary Figure 2: Heatmap showing the correlation among the 7 cell-specific PRSs (*APOE*). A) Heat map of the correlation of cell-specific PRS1. **B)** Heat map of the

correlation of cell-specific PRS2. **C)** Heat map of the correlation of cell-specific PRS3. **D)** Heat map of the correlation of cell-specific PRS4. **E)** Heat map of the correlation of cell-specific PRS5. **F)** Heat map of the correlation of cell-specific PRS6. **G)** Heat map of the correlation of cell-specific PRS7. Corr = Correlation Coefficient generated using the Pearson method. Each data point shows the correlation coefficient value between the two cell-specific PRSs.

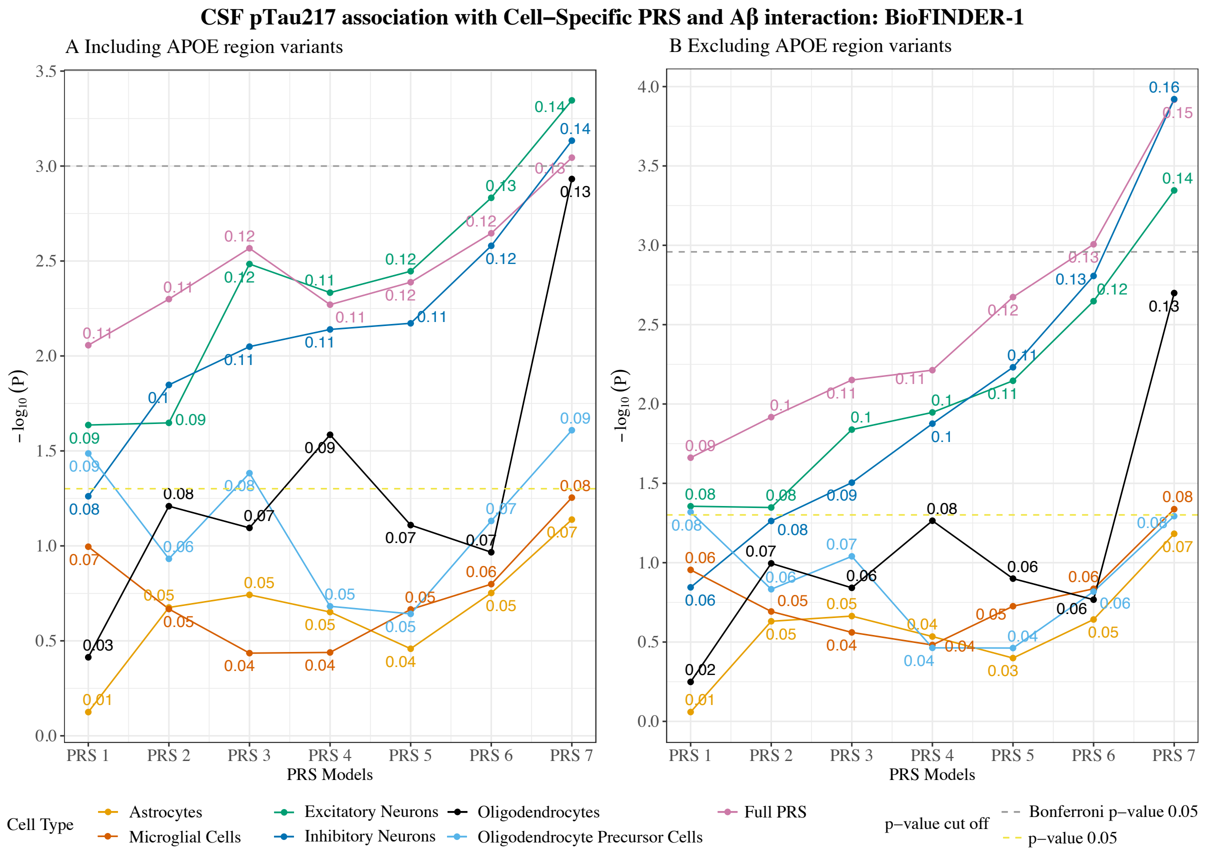

Supplementary Figure 3: Cell-specific PRS associations with CSF pTau217 adjusting for the Aβ x PRS interaction in BioFINDER-1. **A)** Cell-specific PRS associations with CSF pTau217, adjusting for the Aβ x PRS interaction for PRS models generated when including the *APOE* region variants. **B)** Cell-specific PRS associations with CSF pTau217, adjusting for the Aβ x PRS interaction for PRS models generated when excluding the *APOE* region variants. The X-axis represents the respective cell-specific PRS models, and the Y-axis represents the negative log of the p-value for the association. The β-coefficient for the association is given at the top of each point. The models were adjusted for age, sex, Aβ status, Aβ*PRS interaction term and the top 10 genetic principal components. PRS models excluding the *APOE* region variants were additionally adjusted for *APOE* ε4 and ε2 counts. P-value threshold: PRS1 => 0.05, PRS2 => 5e-03, PRS3 => 5e-04, PRS4 => 5e-05, PRS5 => 5e-06, PRS6 => 5e-07, PRS7

=> 5e-08. N = 1293. Linear regression β and p-values are used to plot the figures. Each data point represents a -$\log_{10}$ p-value of the respective PRSs.

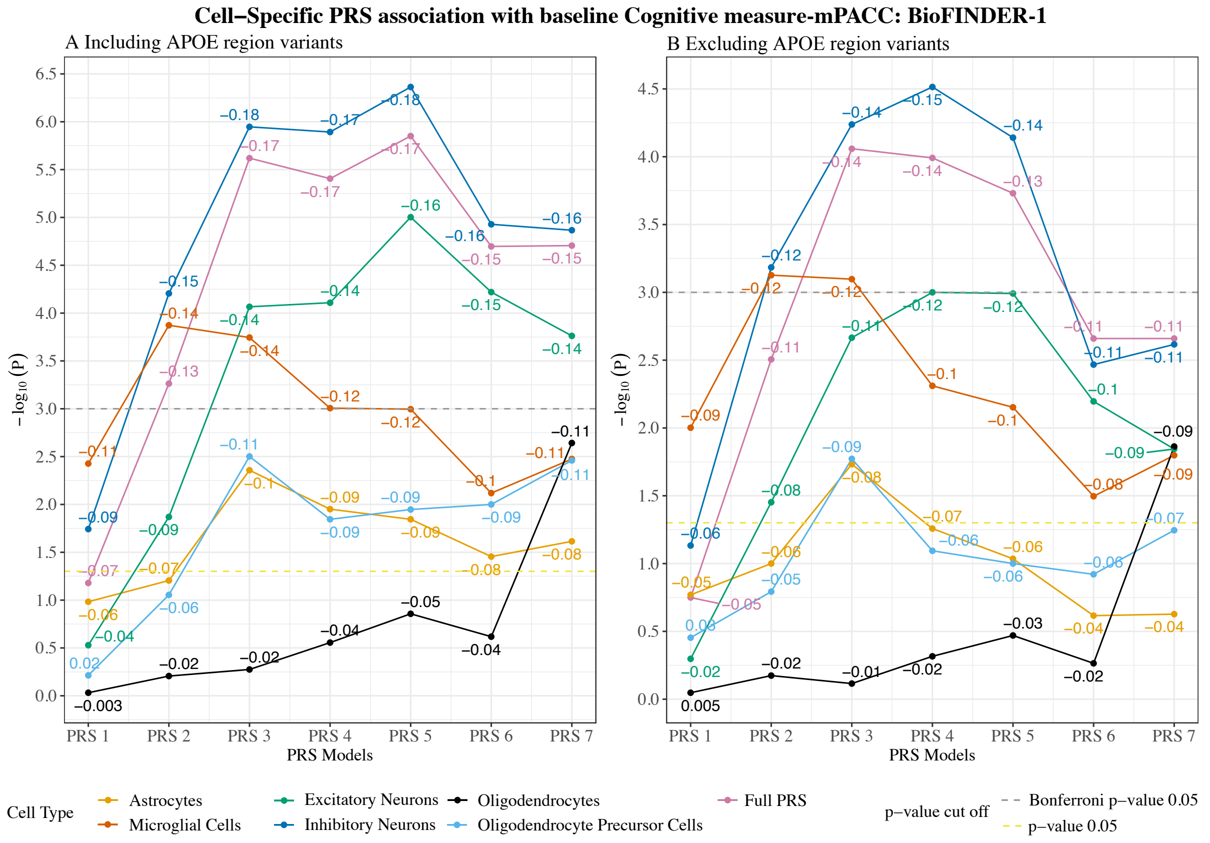

Supplementary Figure 4: Cell-Specific PRS association with baseline cognitive measure (mPACC) in BioFINDER-1. **A)** Cell-specific PRS association with mPACC for PRS models generated when including the *APOE* region variants. **B)** Cell-specific PRS association with mPACC for PRS models generated when excluding the *APOE* region variants. The X-axis represents the respective cell-specific PRS models, and the Y-axis represents the negative log of the p-value for the association. The β-coefficient for the association is given on the top of each bar. The models were adjusted for age, sex and the top 10 genetic principal components. PRS models excluding the *APOE* region variants were additionally adjusted for *APOE* ε4 and ε2 counts. P-value threshold: PRS1 => 0.05, PRS2 => 5e-03, PRS3 => 5e-04, PRS4 => 5e-05, PRS5 => 5e-06, PRS6 => 5e-07, PRS7 => 5e-08. N = 1293. Linear regression β and p-values are used to plot the figures. Each data point represents a -$\log_{10}$ p-value of the respective PRSs.

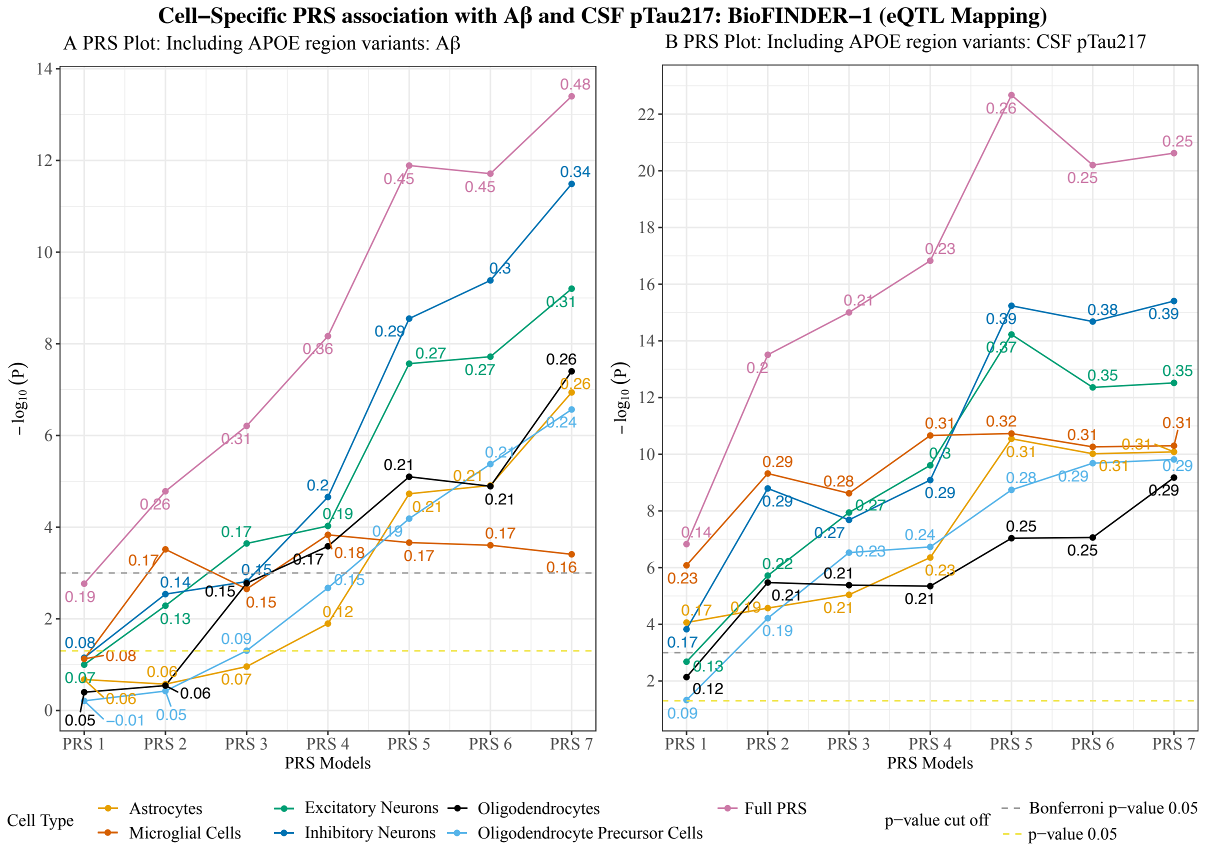

Supplementary Figure 5: Cell-Specific PRS association with Aβ status and CSF p-Tau in BioFINDER-1 using eQTL mapping. **A)** Cell-weighted PRS association with Aβ status for PRS models generated when including the *APOE* region variants. **B)** Cell-weighted PRS association with CSF pTau217 for PRS models generated when including the *APOE* region variants. The X-axis represents the respective cell-specific PRS models, and the Y-axis represents the negative log of the p-value for the association. The β-coefficient for the association is given on the top of each bar. The models were adjusted for age, sex and the top 10 genetic principal components. PRS models excluding the *APOE* region variants were additionally adjusted for *APOE* ε4 and ε2 counts. P-value threshold: PRS1 => 0.05, PRS2 => 5e-03, PRS3 => 5e-04, PRS4 => 5e-05, PRS5 => 5e-06, PRS6 => 5e-07, PRS7 => 5e-08. N = 1293. Linear regression β and p-values are used to plot the figures. Each data point represents a $-\log_{10}$ p-value of the respective PRSs.

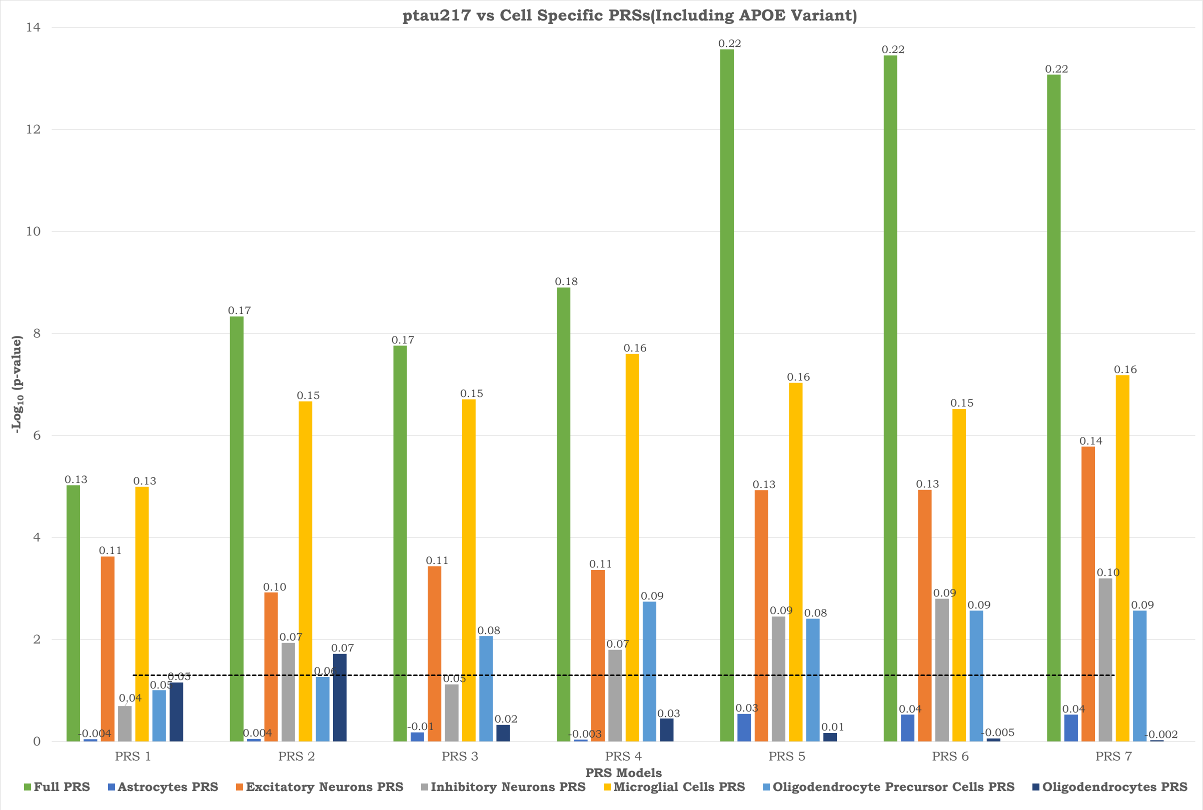

Supplementary Figure 6 A): Cell-weighted PRS association with CSF pTau217 for restricted PRS only to include genes with strict cell-specific expression generated when including the *APOE* region variants. The X-axis represents the respective cell-weighted PRS models, and the Y-axis represents the negative log of the p-value for the association. The β-coefficient for the association is given at the top of each point. The models were adjusted for age, sex and the top 10 genetic principal components. P-value threshold: PRS1 => 0.05, PRS2 => 5e-03, PRS3 => 5e-04, PRS4 => 5e-05, PRS5 => 5e-06, PRS6 => 5e-07, PRS7 => 5e-08. N = 1293. Linear regression β and p-values are used to plot the figures. Each bar represents a -$\log_{10}$ p-value of the respective PRSs.

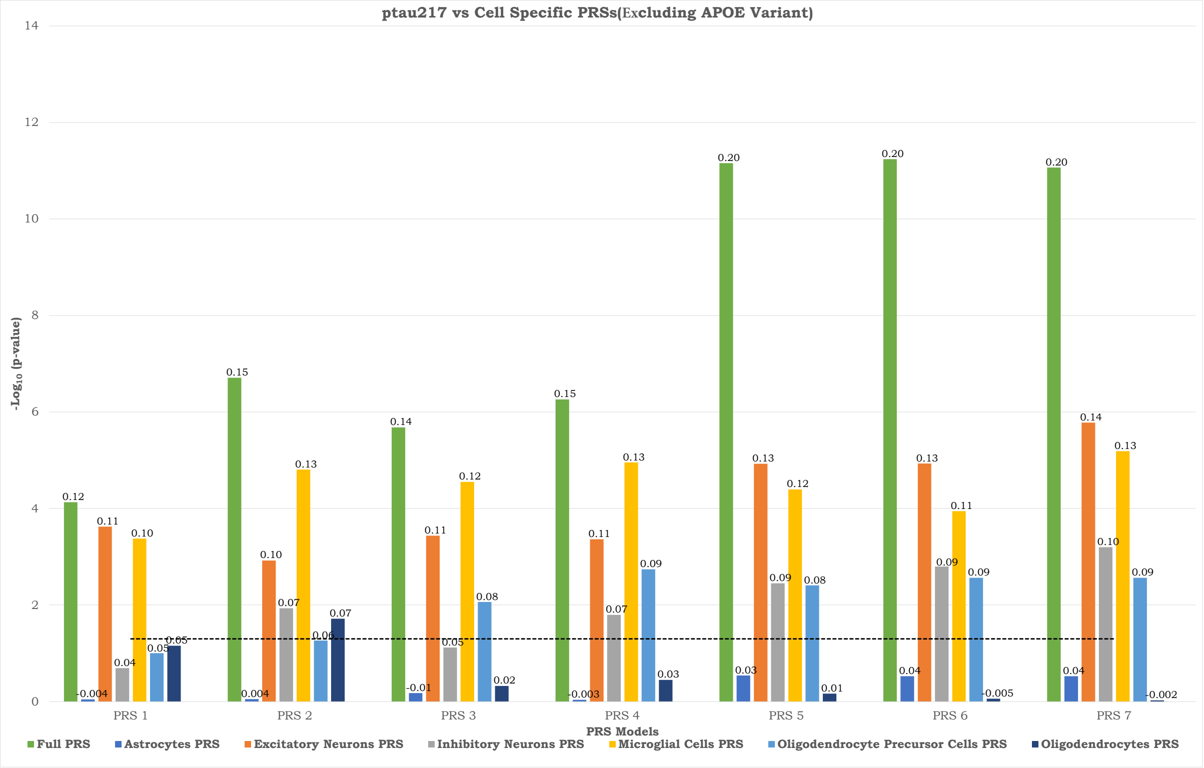

Supplementary Figure 6 B): Cell-weighted PRS association with CSF pTau217 for restricted PRS only to include genes with strict cell-specific expression, generated when excluding the *APOE* region variants. The X-axis represents the respective cell-weighted PRS models, and the Y-axis represents the negative log of the p-value for the association. The β-coefficient for the association is given at the top of each point. The models were adjusted for age, sex and the top 10 genetic principal components. PRS models excluding the *APOE* region variants were additionally adjusted for *APOE* ε4 and ε2 counts. P-value threshold: PRS1 => 0.05, PRS2 => 5e-03, PRS3 => 5e-04, PRS4 => 5e-05, PRS5 => 5e-06, PRS6 => 5e-07, PRS7 => 5e-08. N = 1293. Linear regression β and p-values are used to plot the figures. Each bar represents a $-\log_{10}$ p-value of the respective PRSs.

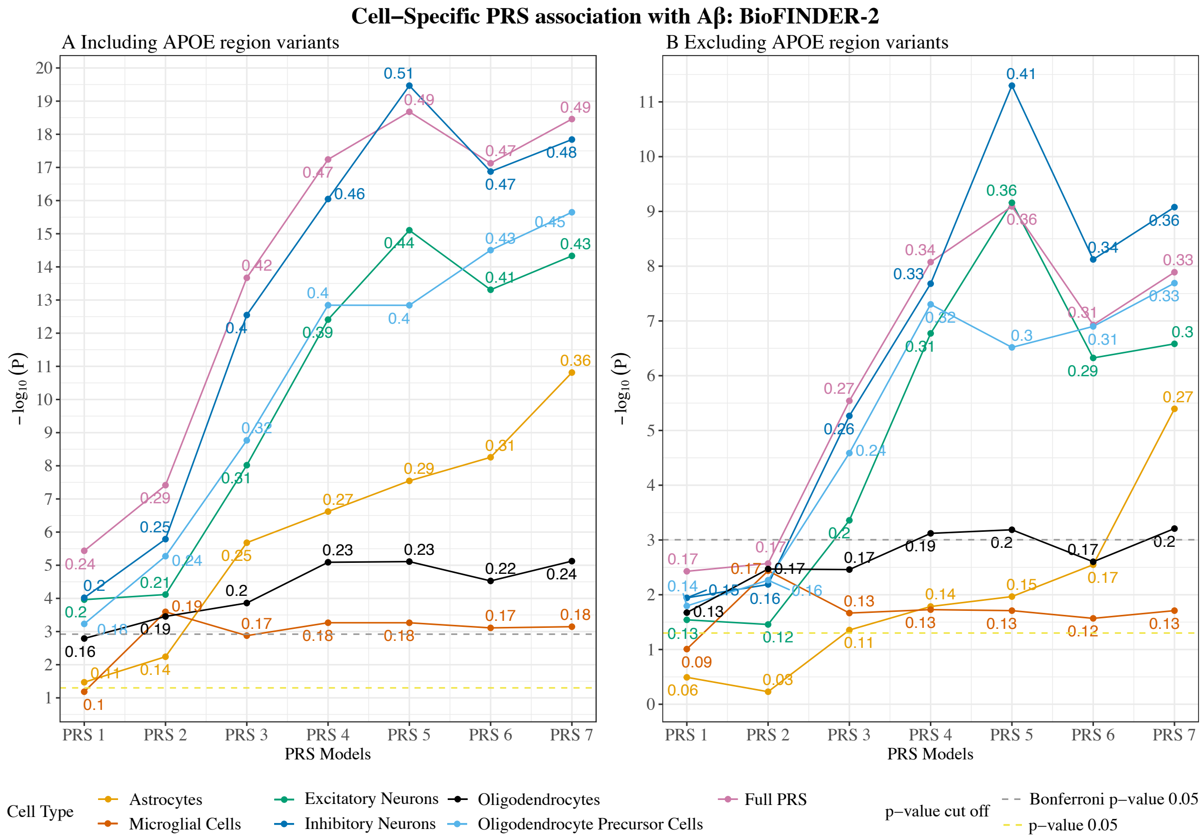

Supplementary Figure 7: Cell-Specific PRS association with Aβ status in BioFINDER-2. **A)** Cell-specific PRS association with Aβ status for PRS models generated when including the *APOE* region variants. **B)** Cell-specific PRS association with Aβ status for PRS models generated when excluding the *APOE* region variants. The X-axis represents the respective cell-specific PRS models, and the Y-axis represents the negative log of the p-value for the association. The β-coefficient for the association is given at the top of each point. The models were adjusted for age, sex and the top 10 genetic principal components. PRS models excluding the *APOE* region variants were additionally adjusted for *APOE* ε4 and ε2 counts. P-value threshold: PRS1 => 0.05, PRS2 => 5e-03, PRS3 => 5e-04, PRS4 => 5e-05, PRS5 => 5e-06, PRS6 => 5e-07, PRS7 => 5e-08. N = 1736. Linear regression β and p-values are used to plot the figures. Each data point represents a -$\log_{10}$ p-value of the respective PRSs.

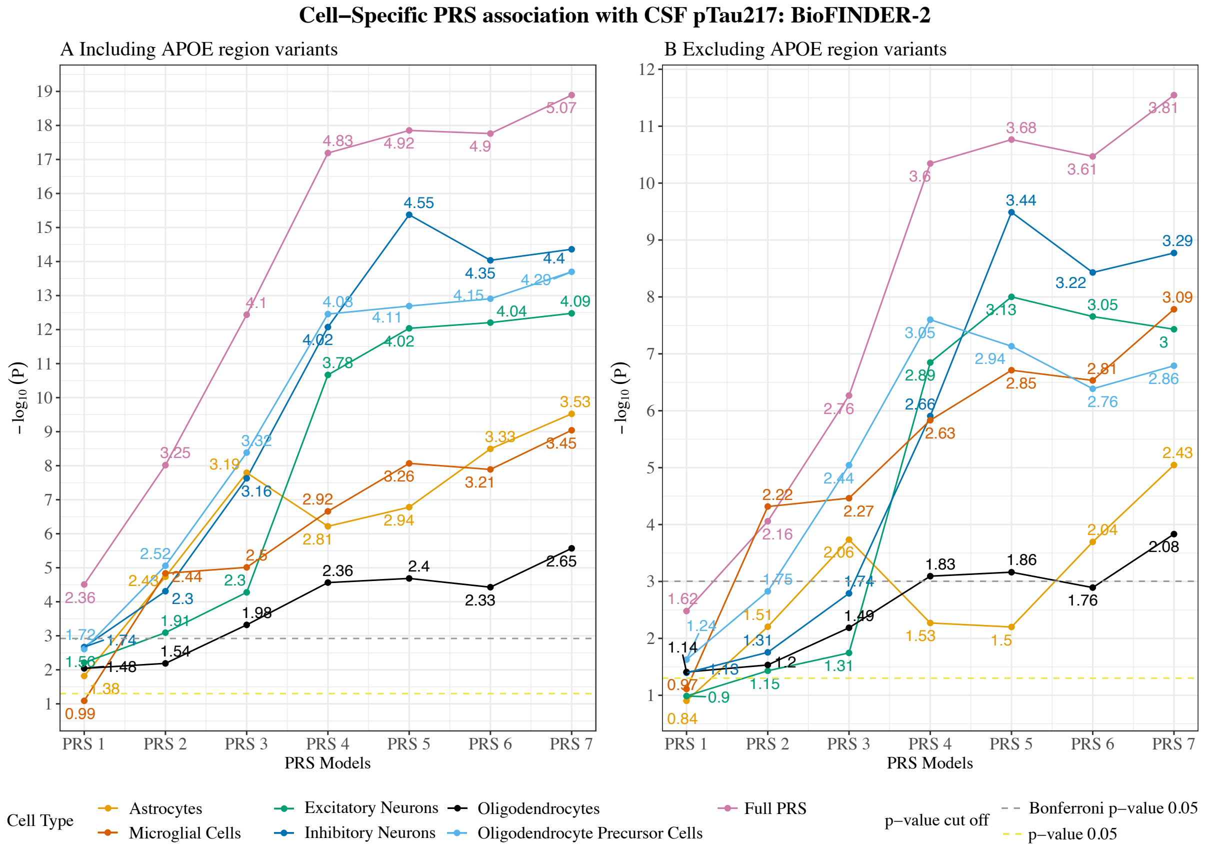

Supplementary Figure 8: Cell-Specific PRS association with CSF pTau217 in BioFINDER-2. **A)** Cell-Specific PRS association with CSF pTau217 for PRS models generated when including the *APOE* region variants. **B)** Cell-specific PRS association with CSF pTau217 for PRS models generated when excluding the *APOE* region variants. The X-axis represents the respective cell-specific PRS models, and the Y-axis represents the negative log of the p-value for the association. The β-coefficient for the association is given at the top of each point. The models were adjusted for age, sex and the top 10 genetic principal components. PRS models excluding the *APOE* region variants were additionally adjusted for *APOE* ε4 and ε2 counts. P-value threshold: PRS1 => 0.05, PRS2 => 5e-03, PRS3 => 5e-04, PRS4 => 5e-05, PRS5 => 5e-06, PRS6 => 5e-07, PRS7 => 5e-08. N = 1736. Linear regression β and p-values are used to plot the figures. Each data point represents a -$\log_{10}$ p-value of the respective PRSs.

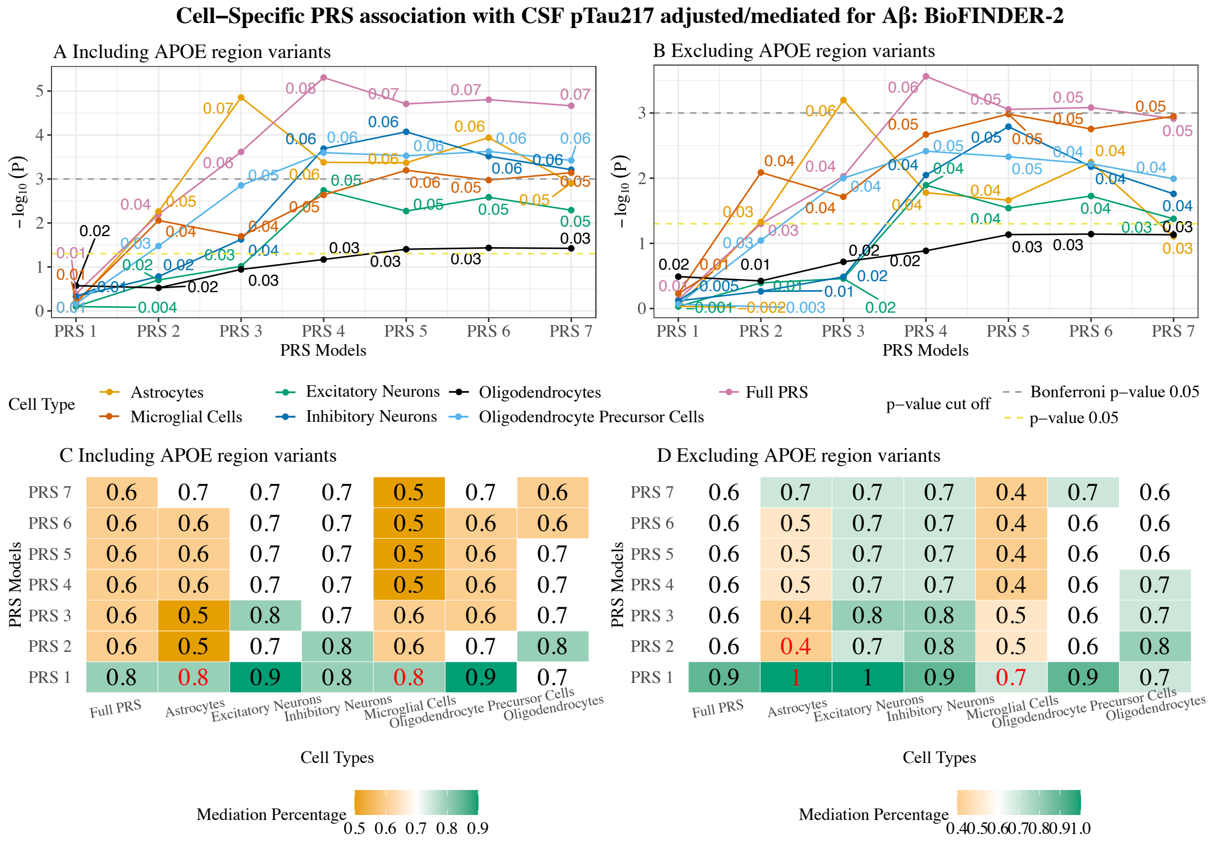

Supplementary Figure 9: Cell-Specific PRS association with CSF pTau217 adjusted/mediated for Aβ status in BioFINDER-2. **A)** Cell-specific PRS association with CSF pTau217 adjusted for Aβ status for PRS models generated when including the *APOE* region variants. **B)** Cell-specific PRS association with CSF pTau217 adjusted for Aβ status for PRS models generated when excluding the *APOE* region variants. The X-axis represents the respective cell-specific PRS models, and the Y-axis represents the negative log of the p-value for the association. The β-coefficient for the association is given at the top of each point. The models were adjusted for age, sex, Aβ status, and the top 10 genetic principal components. PRS models excluding the *APOE* region variants were additionally adjusted for *APOE* ε4 and ε2 counts. P-value threshold: PRS1 => 0.05, PRS2 => 5e-03, PRS3 => 5e-04, PRS4 => 5e-05, PRS5 => 5e-06, PRS6 => 5e-07, PRS7 => 5e-08. Linear regression β and p-values are used to plot the figures. Each data point (for A and B) represents a -$\log_{10}$ p-value of the respective PRSs. **C)** Heat map of Aβ mediation for CSF pTau217 association with PRS models generated when including the *APOE* region variants. **D)** Heat map of Aβ mediation for CSF pTau217 association with PRS models generated when excluding the *APOE* region variants. The X-axis represents the different cell types, and the Y-axis represents the PRS models for respective cell types. The non-significant percentage of mediation is highlighted in red. N = 1736. Each data

point (for C and D) shows the mediation percentage of Aβ for CSF pTau217 association with PRS.

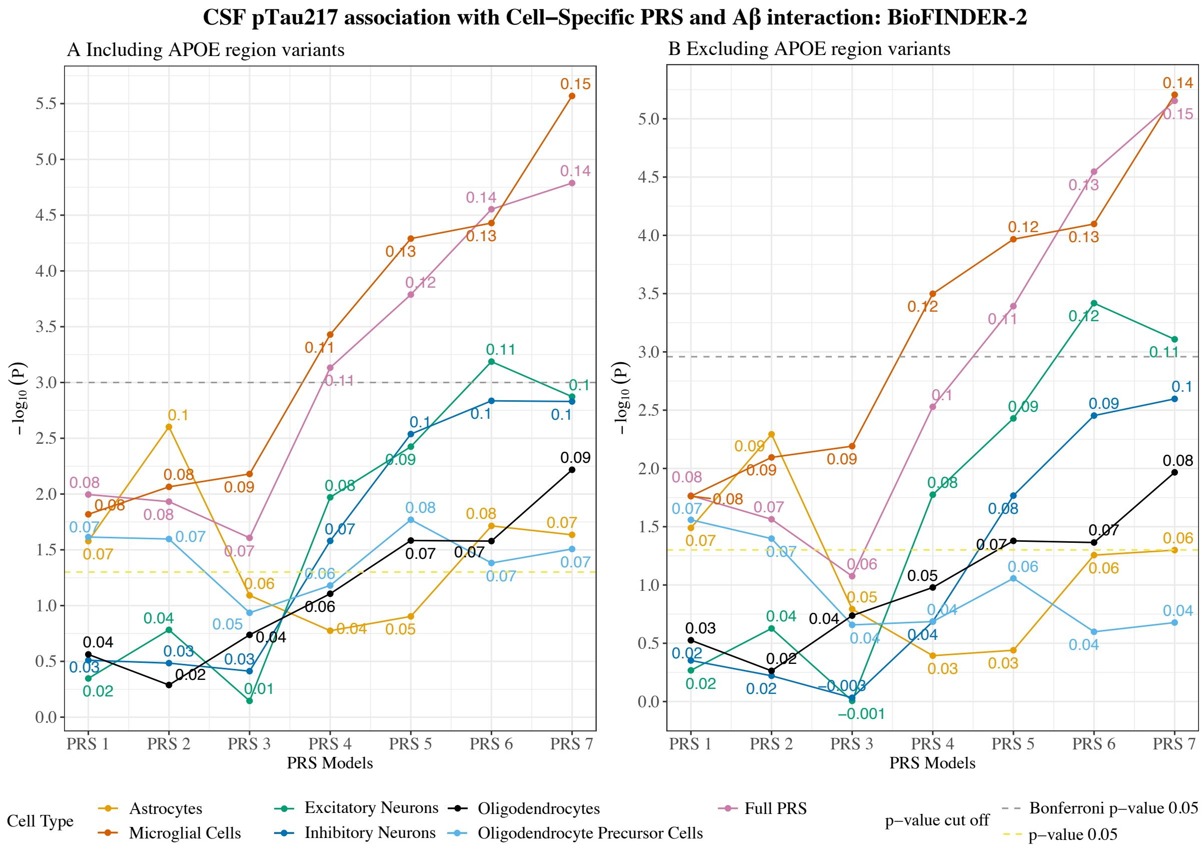

Supplementary Figure 10: Cell-specific PRS associations with CSF pTau217 adjusting for the Aβ x PRS interaction in BioFINDER-2. **A)** Cell-specific PRS associations with CSF pTau217 adjusting for the Aβ x PRS interaction for PRS models generated when including the *APOE* region variants. **B)** Cell-specific PRS associations with CSF pTau217 adjusting for the Aβ x PRS interaction for PRS models generated when excluding the *APOE* region variants. The X-axis represents the respective cell-specific PRS models, and the Y-axis represents the negative log of the p-value for the association. The β-coefficient for the association is given at the top of each point. The models were adjusted for age, sex, Aβ status, Aβ*PRS interaction term and the top 10 genetic principal components. PRS models excluding the *APOE* region variants were additionally adjusted for *APOE* ε4 and ε2 counts. P-value threshold: PRS1 => 0.05, PRS2 => 5e-03, PRS3 => 5e-04, PRS4 => 5e-05, PRS5 => 5e-06, PRS6 => 5e-07, PRS7 => 5e-08. N = 1736. Linear regression β and p-values are used to plot the figures. Each data point represents a -$\log_{10}$ p-value of the respective PRSs.
